# Shift in polar benthic community structure in a fast retreating glacial area of Marian Cove, West Antarctica

**DOI:** 10.1038/s41598-020-80636-z

**Published:** 2021-01-08

**Authors:** Hanna Bae, In-Young Ahn, Jinsoon Park, Sung Joon Song, Junsung Noh, Hosang Kim, Jong Seong Khim

**Affiliations:** 1 School of Earth and Environmental Sciences and Research Institute of Oceanography, Seoul National University, Seoul, 08826 Republic of Korea; 2 Division of Ocean Sciences, Korea Polar Research Institute, Incheon, 21990 Republic of Korea; 3 Department of Convergence Study on the Ocean Science and Technology and Department of Ocean Science, Korea Maritime and Ocean University, Busan, 49112 Republic of Korea

**Keywords:** Climate-change ecology, Ecosystem ecology, Marine biology

## Abstract

Glacier retreat is a major long-standing global issue; however, the ecological impacts of such retreats on marine organisms remain unanswered. Here, we examined changes to the polar benthic community structure of “diatoms” under current global warming in a recently retreated glacial area of Marian Cove, Antarctica. The environments and spatiotemporal assemblages of benthic diatoms surveyed in 2018–2019 significantly varied between the intertidal (tidal height of 2.5 m) and subtidal zone (10 and 30 m). A distinct floral distribution along the cove (~ 4.5 km) was characterized by the adaptive strategy of species present, with chain-forming species predominating near the glacier. The predominant chain-forming diatoms, such as *Fragilaria striatula* and *Paralia* sp., are widely distributed in the innermost cove over years, indicating sensitive responses of benthic species to the fast-evolving polar environment. The site-specific and substrate-dependent distributions of certain indicator species (e.g., *F. striatula*, *Navicula glaciei*, *Cocconeis* cf. *pinnata*) generally reflected such shifts in the benthic community. Our review revealed that the inner glacier region reflected trophic association, featured with higher diversity, abundance, and biomass of benthic diatoms and macrofauna. Overall, the polar benthic community shift observed along the cove generally represented changing environmental conditions, (in)directly linked to ice-melting due to the recent glacier retreat.

## Introduction

The climate crisis is highlighted by the breaking of temperature records during the austral summer in the Antarctic continent especially in West Antarctica^1^, and the ecological impacts of these changes becoming increasingly serious^2,3^. Glacier retreat is reported to cause rapid and severe impacts on both plankton and benthos by melt-water and drifting ice^4^. Glacier retreat together with ice-melt in the fjords of West Antarctica is also expected to affect benthic microflora, leading to blooms of seabed diatoms^5^. However, the ecological influence of glacier retreat on the spatial development and/or year-round variation of benthic diatoms remains largely unknown.

Diatoms are important primary producers in Antarctica^6^, and are used as biological indicators of the rapidly changing environment, such as global warming^7,8^. Despite their ecological significance in the coastal waters of Antarctica, response of polar benthic diatoms to glacier retreat remains limited understanding. Given that shallow coastal waters in Antarctica will likely be impacted by anticipated glacial melting, it is necessary to obtain a sound ecological understanding of benthic diatoms.

**Figure 1:**
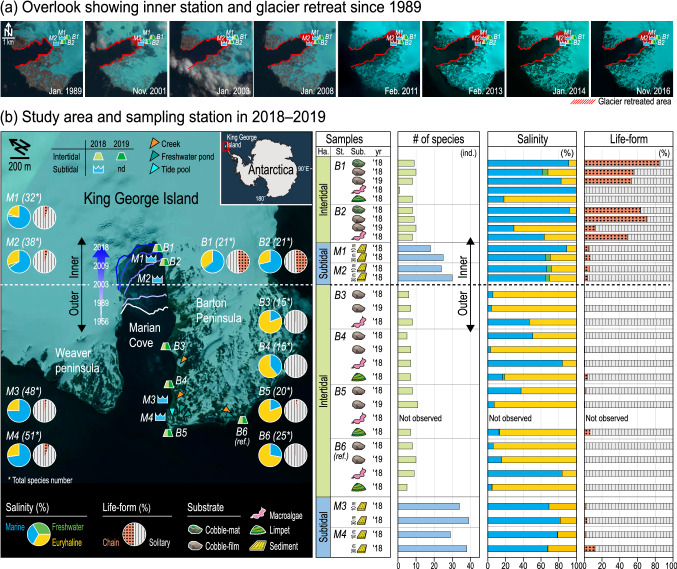
Overview of the study area, sampling locations, and benthic diatom community data; (**a**) Overlook showing glacier retreat in Marian Cove, West Antarctica, since 1989. Stations in the inner part of the cove are presented (B1, B2, M1, and M2). (**b**) Map showing sampling locations (intertidal zone: B1–B6; subtidal zone: M1–M4 at 10 and 30 m) surveyed in 2018–2019, with a summary of benthic community data; specifically, the number of species, relative abundance by salinity (marine, euryhaline, and freshwater), life-form (chain and solitary), and various substrates (cobble-mat, cobble-film, macroalgae, limpet shell, and sediment). Ecological type of diatom species in relation to salinity is provided in Table S8. The base map was obtained from Google Earth (https://earth.google.com/web) and Global USGS Visualization Viewer (https://glovis.usgs.gov).

Here, we examined changes to the polar benthic community structure of diatoms under current global warming in a recently retreated glacial area of Marian Cove, West Antarctica (Fig. 1). In specific, we aimed to: (1) evaluate the overall diversity of benthic diatoms in intertidal and subtidal habitats, (2) characterize composition of species and their distribution characteristics with respect to the types of substrates that they inhabit, and (3) address early ecological responses of polar benthos and their adaptive strategies, by integrating the previous results (a mini-review) into the present findings. The results of the current study are expected to provide reference data on how the retreat of glaciers is affecting marine ecosystems in the rapidly changing and harsh environment of the polar region.

## Materials and methods

### Study area

The South Shetland Islands encompass 11 mountainous islands situated ~ 160 km north of the Antarctic Peninsula. King George is the largest island of these islands. The study area is one of two large fjords on King George, located on the Barton Peninsula, West Antarctica (Fig. S1). Two tributary basins, Marian and Potter Cove, are situated on either side of Barton Peninsula. The present study focused on the marine benthic ecosystem of Marian Cove, which shows distinct environmental gradients caused by glacial melting during the austral summer^9^. As glacier retreated, the environment on the inner cove has become more deepen bay system, leading to provide relatively stable condition in physical stress such as wave, wind, current, and ice scouring compared to open shore^10–12^.

### Sampling and laboratory analyses

Sampling was conducted at a total of 14 locations, encompassing intertidal locations (*n* = 6; B1–B6) and subtidal locations at two depths (*n* = 8; M1_10m_–M4_10m_ and M1_30m_–M4_30m_) during the austral summer of 2018 and 2019 (Fig. 1). All of the intertidal and subtidal locations were surveyed simultaneously in 2018, and six locations (B1–B6) from the intertidal zone were surveyed again in 2019, with the specific aim of investigating year-round changes to the intertidal benthic community. Out of the intertidal locations, five (B1–B5) were situated along the coast of Marian Cove, while B6, a reference site, was located at the southern part of Barton Peninsula. Of note, B1 and B2 were situated in a recent glacier retreat zone, and were approximately 100 and 360 m distant from the glacier in Marian Cove, respectively, at the time of sampling in 2018.

Seawater properties were measured in situ using a multi-parameter water quality probe (YSI-Professional plus, USA), and included temperature (°C), dissolved oxygen (DO, mg L^−1^), salinity (psu), pH, and SiO_2_ (μg L^−1^). Total phosphorus (TP, μg L^−1^) and nitrogen (TN, μg L^−1^) were measured following standard methods^13^. Waterborne particulate organic matter (POM) and diatom samples were lyophilized before stable isotope analysis. Concentrations of the stable isotopes of carbon (δ^13^C) and nitrogen (δ^15^N) in POM and diatom samples were measured using an Elemental Analyzer-Isotope Ratio Mass Spectrometer (EA-IRMS; Elementar, Gmbh, Hanau, Germany). High purity carbon dioxide and nitrogen were used as reference gases, while helium and oxygen were used as carrier and combustion gases. Stable carbon and nitrogen isotopic compositions were expressed as ‰ delta notation (Eq. 1):

1 $$\delta^{{{13}}} {\text{C or }}\delta^{{{15}}} {\text{N }}\left( \permil \right) \, = \, \left[ {{\text{R}}_{{{\text{sam}}}} /{\text{R}}_{{{\text{ref}}}} {-}{ 1}} \right] \, \times { 1}000$$

where R_sam_ and R_ref_ are the compositions (^13^C/^12^C or ^15^N/^14^N) of the sample and reference, respectively. Isotopic compositions were reported relative to conventional reference materials; specifically, Vienna Peedee Belemnite (VPDB) for carbon, and atmospheric N_2_ for nitrogen. IAEA-CH-3 and IAEA-N-2, which are international isotope standards, were used as reference materials to calculate the analytical error of carbon and nitrogen, respectively. Measurement precision was approximately 0.04‰ for δ^13^C and 0.2‰ for δ^15^N.

Diatom assemblages were collected from three different substrates in the intertidal zone (*n* = 24; viz., cobble, macroalgae, and *Nacella concinna* limpet shells) and one substrate type in the subtidal zone (*n* = 8; bottom sediment, < 1 cm). Of note, two different diatom forms were sampled on cobble surfaces; namely, thick carpet-like mat (cobble-mat) and thin biofilm (cobble-film) (Fig. S1). The only macrofaunal organism found in high numbers in the intertidal zone was the limpet *N. concinna*, the shell of which forms a habitat for diatoms. All the intertidal samples had been collected when the sites are exposed to the air during low tide.

Diatoms collected from intertidal zone were removed from the surface of substrates with a toothbrush. Sampling was conducted with three replicates of samples (cobbles, fronds of macroalgae and limpet shells). The top 1.0 cm of the surface sediment was collected from the subtidal zone by scuba divers. Three-hundred diatom valves per sample were identified and counted, in most cases, to calculate relative abundance. Of note, limited numbers of diatoms were attached to macroalgae at locations B1 and B5 (only 0–2 valves were observed). Diatoms were classified to the species level where possible. Concentrated solutions of HCl and H_2_O_2_ were used to remove calcium carbonate particles from sediment and organic material from cells, respectively. Permanent slides were made using Naphrax resin. A light microscope (Olympus BX53) and scanning electron microscope (Tescan MIRA-3) were used to obtain photographic documentation. Photographs of dominant diatoms are presented in Fig. S2–S8.

### Data analysis

Data were analyzed statistically, as summarized in Table S1. Indicator value analysis (IndVal) was performed to identify indicator diatom species within each group following cluster analysis^14^. This was completed to identify indicator species that were linked to corresponding geographical features of habitats. Of note, among the types of intertidal substrates, epilithic diatoms are targeted. These diatoms would minimize bias due to substrate-dependent variation.

## Results

### Environments

The salinity of the study area showed no clear spatiotemporal variation and/or trends during the survey (Table S2). However, freshwater input to the cove was occasionally observed from the glacier and creeks at the time of sampling. To identify terrestrial influence on the cove, concentrations of δ^13^C and δ^15^N in the POM of seawater and benthic diatoms were measured along the cove (B2, B4–B6; insufficient samples were available for B1 and B3). The most enriched δ^13^C concentrations of POM (− 18.2‰) (size-fractionated at 20–100 μm reflecting microplankton size) and diatoms (− 16.6‰) were detected in the inner cove (B2), near the glacier. Also, the δ^13^C values of POM and diatoms tended to decrease with increasing distance from the glacier (− 24.1 to − 18.2‰ and − 23.2 to − 16.6‰, respectively). The δ^15^N values of POM and diatoms showed no clear spatial trend. The concentrations of total nitrogen did not vary across the locations (mean = 0.8 µmol L^−1^), but reference location B6 at the Narebski point, where large penguin colonies developed, showed the elevated TN (1.8 µmol L^−1^).

### Assemblages of benthic diatoms

A total of 92 diatom taxa were recorded and identified from all surveyed locations during the study period (Table S3–S5). In general, the community structure of benthic diatoms showed high spatial variation, but low temporal variation between 2018 and 2019 (Fig. 1b). Significant differences in the floral diversity were observed between inner and outer region, which was supported by the results of the stable isotopic analyses (Table S2). In particular, the diversity of subtidal benthic diatoms almost doubled in the outer locations (M3–M4) compared to the inner locations (M1–M2). Benthic community structure between regions and/or locations lacked similarity, based on species compositions associated with ecological type and/or life-form^15^. For example, most diatoms were solitary, but chain-forming diatoms dominated in the inner intertidal zone of the Marine Cove, and had the most distinct distribution characteristics of polar benthic diatoms near the glacier.

Out of the 49 diatom taxa identified in the intertidal zone, *Navicula* cf. *perminuta* (53.5%) and *Fragilaria striatula* (14.4%) were the two predominant species. In comparison, chain-forming diatoms, such as *F. striatula*, *F.* cf. *striatula*, and *Fragilaria islandica* var. *adeliae*, dominated (48.0%) the inner intertidal locations (B1–B2).

Subtidal benthic diatoms included 82 taxa, which exhibited higher diversity compared to intertidal benthic diatoms, despite substrate type being limited to muddy bottoms. Dominant species were *N.* cf. *perminuta* (16.7%) and *Navicula glaciei* (15.9%). Of note, *N.* cf. *perminuta* appeared across all locations in Marian Cove. In comparison, the diatom, *N. glaciei*, dominated M1 and M2 (76.7% and 37.0%, respectively), and did not occur in the outer locations (M3–M4).

### Distribution characteristics of benthic diatoms

**Figure 2:**
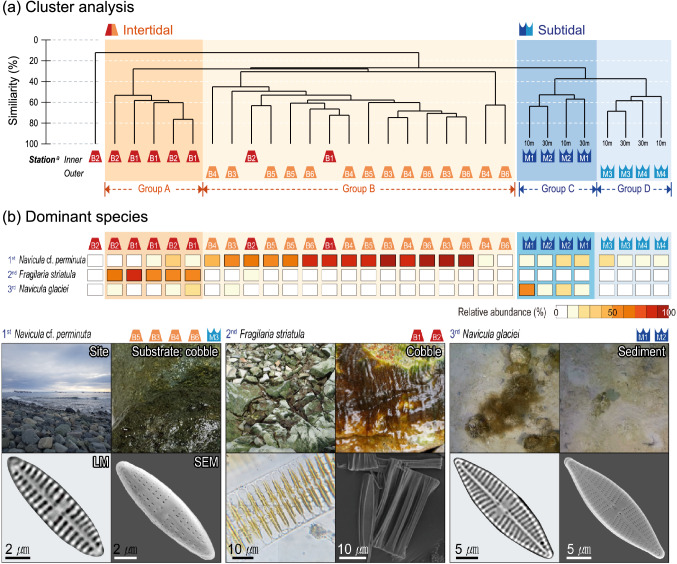
Community structure of benthic diatoms in Marian Cove, West Antarctica; (**a**) cluster analysis results showing the four groups of diatom assemblages (^a^Sampling details given in Fig. S1). (**b**) Relative abundance of the top three dominant species in the corresponding locations with site/substrate view and LM and SEM images.

**Figure 3:**
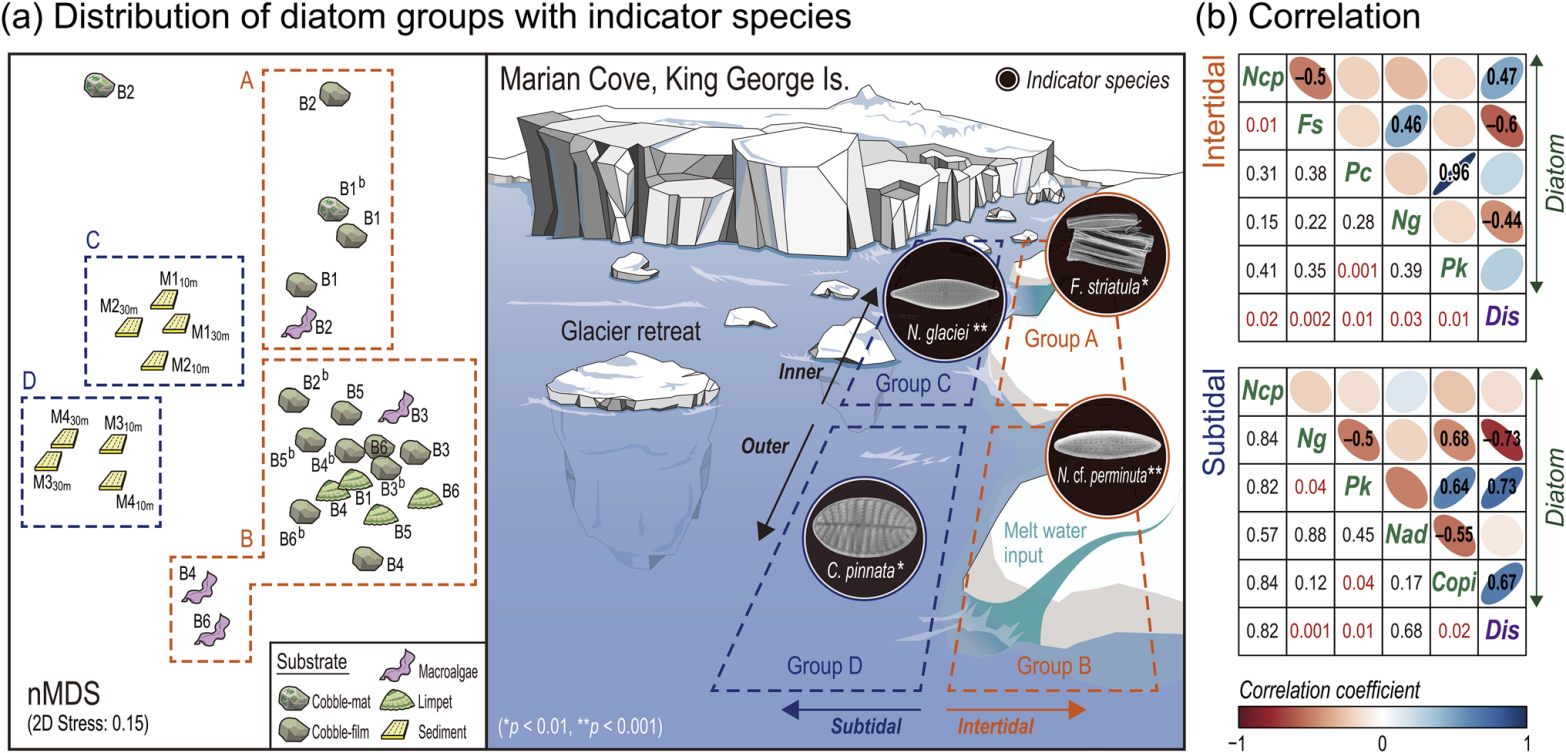
Four indicator species of each environment in Marian Cove, and the correlation between species and distance from the glacier; (**a**) Diagram of the study area with indicator diatom species (SEM images) along the cove and nMDS ordination plot based on relative abundance. (**b**) Correlation between top five dominant species in each intertidal and subtidal zone (Ncp, *Navicula* cf. *perminuta*; Fs, *Fragilaria striatula*; A3, *Achnanthes* sp. 3; N1, *Navicula glaciei*; Pk, *Pseudogomphonema kamtschaticum*; Nad, *Navicula directa*; Copi, *Cocconeis* cf. *pinnata*) and distance from the glacier (Dis). *p*-values are given as numbers.

Four benthic diatom groups were identified by cluster analysis (ANOSIM: *R* = 0.83, *p* < 0.01), and were separated in respect to: (1) water depth (intertidal and subtidal zones), (2) distance from the glacier, and (3) habitat substrate (Figs. 2, 3, Fig. S9). For intertidal diatoms, inner and outer assemblages were separated as Group A and B, respectively. Two locations (B1–B2) in Group A belonged to the glacier retreat zone (< 1.5 km, ice-free area due to glacier retreat since the 1990s). The dominance of chain-forming diatom, *F. striatula*, was characteristic of Group A. Group B mainly encompassed outer intertidal locations (B3–B6), dominated by diatoms attached to limpet shells. The indicator species of Group B was *N.* cf. *perminuta* (69.2%), which is a commonly occurring species in Marian Cove (Fig. 2b). SIMPER analysis (cut-off 70%) confirmed that *N*. cf. *perminuta* contributed most to the Group B assemblage. Of note, each of the five predominant species from the intertidal and subtidal zones showed positive or negative correlations to distance from the glacier (Fig. 3b).

Subtidal diatom assemblages were also separated by geographical location. The genus *Navicula*, including *N. glaciei* and *N*. cf. *perminuta*, dominated the inner region (M1–M2), and belonged to Group C. *Cocconeis* cf. *pinnata* was the dominant species in Group D (> 10%), which included the outer subtidal locations (M3–M4). When compared to the other groups, this species represented < 2% of the total relative composition of diatoms. Overall, the community structure of subtidal benthic diatoms had lower spatial variation compared to intertidal species. However, certain euryhaline species, such as *N.* cf. *perminuta* and *Achnanthes brevipes* var. *intermedia*, were consistently observed both in the inner and outer cove, regardless of water depth (Table S5). Significant positive correlations were obtained for some dominant species, such as *Pseudogomphonema kamtschaticum*, with other taxa (*p* < 0.05) both in the intertidal and subtidal zones, indicating spatial interactions among certain species (Fig. 3b).

## Discussion

### Impact of glacial retreat on the benthic ecosystem

Most enriched POM δ^13^C concentration in the inner cove location (B2) indicates a potential melt-water input near the glacier (Table S2). The δ^13^C signature of diatoms showed a similar spatial concentration gradient along the cove, but was slightly more enriched than POM δ^13^C. This signature of freshwater influence has also been detected in other Antarctic regions. For example, the enriched δ^13^C of POM and diatoms in Potter Cove was recently reported^16^. In the enclosed environment beneath glaciers, δ^13^C might be enriched due to increased HCO_3_^−^ utilization and production of organic materials^17^. The POM and diatom δ^15^N concentrations showed the lack of parallel gradients over the study area. The POM δ^15^N, especially phytoplankton values, is affected by their nutrient sources. Snow melt-water input occasionally appears from the local creeks throughout the Marian Cove, and the melt-water is associated with the nutrient input as well. Thus, the POM and diatom δ^15^N concentrations seemed to reflect the melt-water input throughout the cove.

The coastline of the inner locations (B1–B2; < 0.5 km to Marian Cove glacier) is covered by snow and ice during winter, and is exposed to the atmosphere during summer. The recent glacier retreats during the 2010s rendered the B1 location ice-free (Fig. 1a). In general, when ice cover melts, a rocky shore is revealed on which diatoms quickly emerge, ultimately attaining considerable biomass^18^. In our study area, a sea ice diatom *F. striatula* covered the inner intertidal rocky shore (B1–B2) like a thick carpet (Fig. 2b). This chain-forming species is likely a rapid colonizer in Marian Cove. Several species belonging to the genus *Fragilaria* have been previously reported as pioneering diatom taxa in ice-melting areas^19^ and estuary^20^. The inner cove environment seemed to stimulate the early aggregation of chain-forming diatoms, indicating the presence of adaptative community responses in glacier retreat zone.

*N.* cf. *perminuta* was the most abundant species at all locations. *N.* cf. *perminuta* also dominated on limpet shells at all locations. Limpets are able to tolerate physical stress under rapid temperature change^21^; thus, *N*. cf. *perminuta* might share and endure the conditions of limpets by settling on the top of shells. The diatoms on limpet shell might also be exposed to the harshest environments. However, the large abundance of limpets in the benthic environment of Marian Cove might represent the best alternative habitat when lacking in soft bottom sediment, on which they were rarely distributed. *N*. cf. *perminuta* is presented in various region of Antarctica including Marian Cove, South Bay, and Ross Sea^5,22,23^. The species has also been reported to dominate across various substrates such as cobble^24^, most of macroalgae^25^, surface of animals^26^, and artificial substrate^22^, although it appeared less on macroalgae in this study area. Thereafter *N*. cf. *perminuta* is considered to be one of the best adapted species in Antarctica*.* The motility of limpets might also explain the broad occurrence of *N.* cf. *perminuta*; however, more information on its ecology is required^27^.

The large numbers of euryhaline diatoms (including *N*. cf. *perminuta*) observed across all the locations indicated the presence of melt-water (freshwater) inputs around Marian Cove. However, the high numbers of marine species in subtidal locations (M1–M4) indicated low freshwater input in the deep waters of the cove (Fig. 1). Species diversity was much greater on the muddy bottoms of subtidal deep waters compared to intertidal substrates. Unlike the intertidal zone where few species dominated (*F*. *striatula* in inner cove (39.5%) and *N*. cf. *perminuta* in outer cove (68.5%)), sedimentary diatoms exhibited relatively high evenness (Table S6). Several diatoms belonging to the genera *Navicula* and *Cocconeis* were widespread subtidal species, occupying a distinct zone to intertidal habitats. Finally, relatively consistent proportions of subtidal diatom assemblages were recorded across all the surveyed locations. This phenomenon implies that thermohaline changes more prevailed by ice-melting and/or physical stress of ice-scouring in the intertidal areas than deep waters, supporting observations that shallow waters are relatively fragile to the effects of melting ice^19^.

### Indicator species

Six indicator species were identified in Marian Cove (*p* < 0.05 in IndVal), with four species being representative of clusters A, B, C, and D, respectively (Fig. 3a, Table S7). Group A inhabited newly exposed ice-free areas, with dominance of *F*. *striatula*, being the indicator species. *F*. *striatula* has been reported as an indicator of cooler temperature with presence of floating sea ice throughout the austral summer^10^. In the meantime, results from the present study suggest that the species could also indicate the influence of broken pieces of floating sea ice which have drifted to the shore. *F. striatula* may have settled down on the intertidal zone after last sea ice melted, subsequently becoming a predominant indicator species as rapid colonizer to the newly exposed ice-free area. The indicator species of Group B was *N*. cf. *perminata*, which occupied the outer intertidal habitats. This species was able to withstand extreme conditions on hard substrate. The indicator species of Group C were Naviculoid diatoms such as *N. glaciei* (sea ice diatom) and *Navicula directa*, which occupied the inner subtidal sediment. *N. glaciei* seemed to be dominated through a similar process to the *F. striatula* in the intertidal zone. The dominated occurrence of *N. glaciei* and *F. striatula* found in the austral summer would reflect the presence of sea ice during the colder season followed by ice-melting at the time of sampling^10^. Of note, some earlier studies have reported the dominance of *N. glaciei* in the subtidal zone around the glacier retreating area^5,22^. Two epiphytic diatoms, *C.* cf. *pinnata*^28^ and *P. kamtschaticum*^29^, were the indicator species of Group D. Although these diatoms inhabited sediment, the abundant epiphytic diatom reflected the available habitats for benthic diatoms in the deep waters of cove. The result was generally consistent with the previous studies that documented prevailed subtidal epiphytes on Antarctic macroalgae^23,25,29^. These abundant macroalgae colonized in the outer subtidal zone, which might represent the preferable habitat for those taxa.

The lack of overlapping indicator species across the groups supported clear distinct of benthic diatom assemblages among the groups. Overall, our analyses revealed the presence of dynamic, sensitive, and distinct micro-benthic community that was responding to ice melting under the rapidly changing polar environment. In fjord-shaped coves, such as Marian Cove in the present study area, the sea ice of the inner part is the last to disappear. Interestingly, both indicator species of the inner part, viz. *F. striatula* and *N. glaciei* were sea ice diatoms, which are released with melting ice during austral spring^10,30^. Considerable abundance of diatoms in the inner cove overlapped that of the sea ice diatom. Previous studies also reported that the sea ice diatoms are released into the water column after the sea ice has melted^22^ and they may settle down on other substrates such as surface of macroalgae^23^. Thus, the high abundance of these species likely reflects a temperature cooling event in the area proximal to the glacier retreat region.

### Role of benthic diatoms on shift in polar community

**Figure 4:**
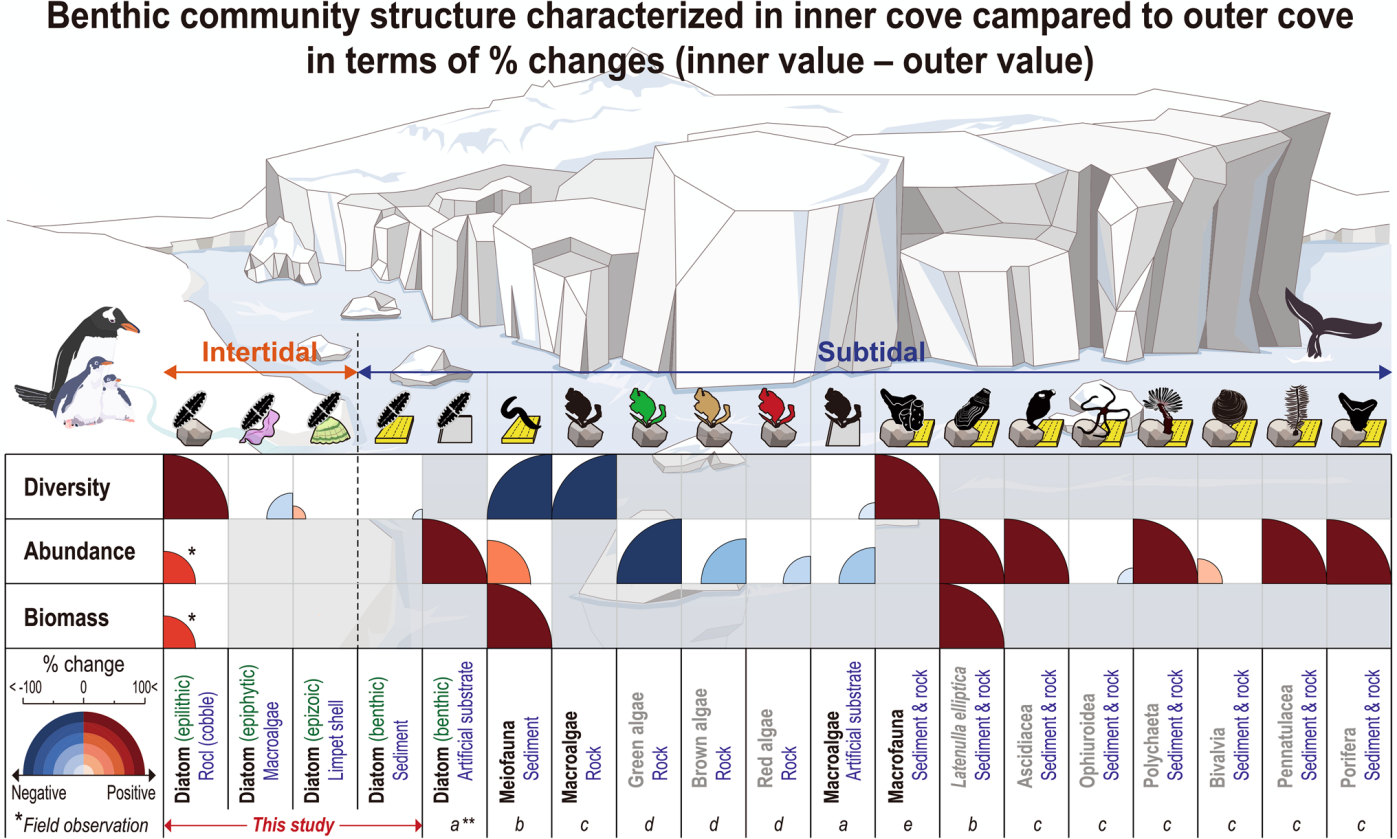
Mini-review on the ecological responses of marine benthic organisms affected by glacier retreat in Antarctica (^*^this study and ^**^five references^a,^^35^^;b,^^37^^;c,^^32^^;d,^^31^^;e,^^40^). Benthic community structure characterized in inner cove compared to outer cove in terms of % changes. % changes in diversity, abundance, and biomass of marine benthos between the inner (< 2.5 km to glacier) and outer (> 2.5 km) region; “positive”  indicates greater value in the inner region than outer one. Target marine organisms include diatoms, meiofauna, macroalgae, and diverse macrofauna.

Studies investigating the ecological responses of polar benthic organisms to glacier retreat remain limited. Our mini-review demonstrated that ecological responses vary depending on the target taxa present (Fig. 4). The diversity and abundance of macroalgae tends to be lower in inner cove^31,32^. In comparison, the diversity and abundance of small organisms, such as meiofauna and diatoms, is higher in the inner cove. During our survey, we documented large benthic diatom blooms in the inner cove (Fig. 2b and Fig. S1b), with previous studies supporting this phenomenon^33,34^. The elevated number of epibiotic diatom species in blooms occurring in the subtidal zone potentially indicate the presence of a mature benthic community that is less influenced by ice-melting events. The phenomenon of enriched massive chain-forming diatoms observed in the subtidal zone of Marian Cove was recently documented^5,33^. Of note, a higher diatom growth near the glacier at the initial phases of experiment using artificial substrates (macroalgae) was documented in Potter Cove, which is adjacent to Marian Cove^35^.

**Figure 5:**
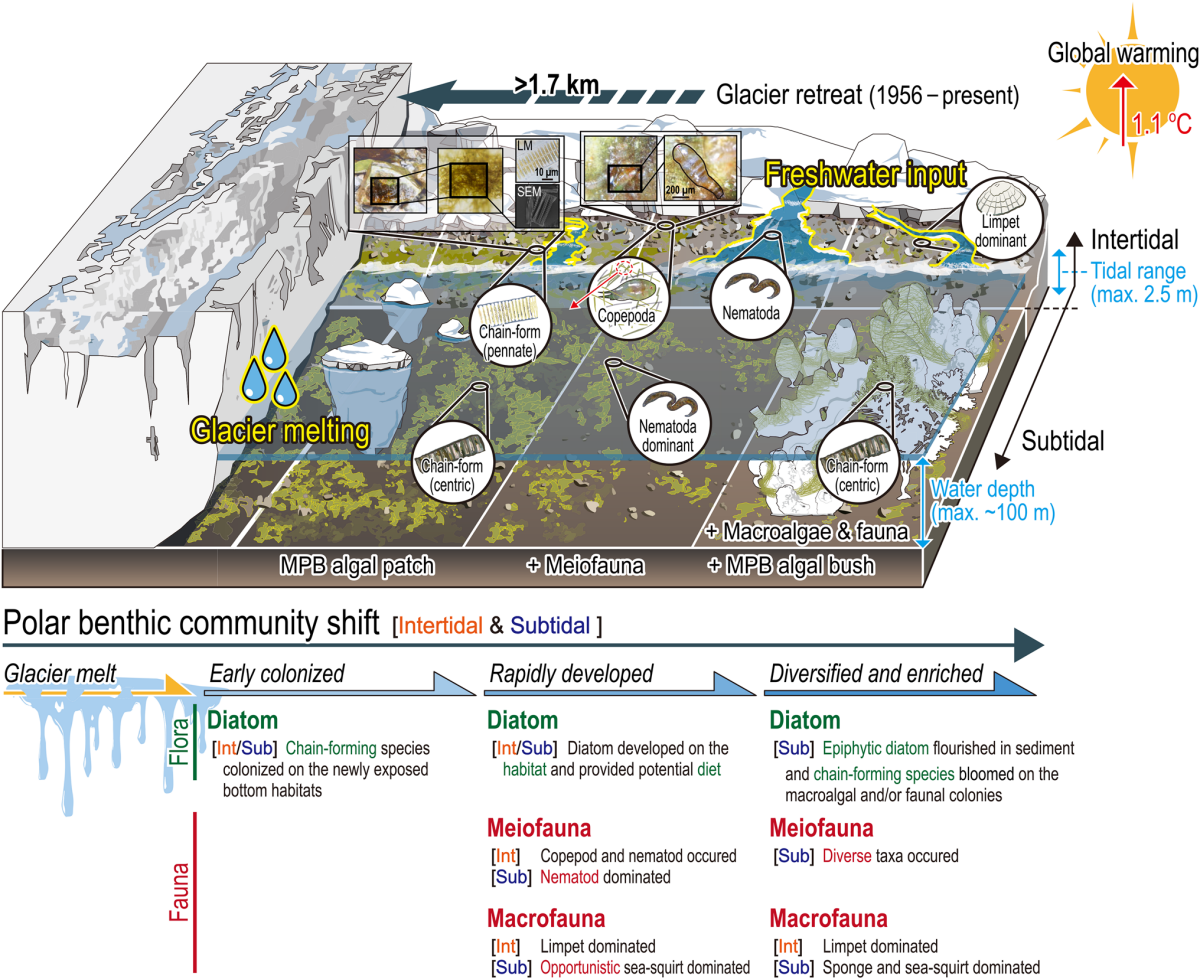
A schematic overview of the polar benthic community shift in an Antarctic cove under glacier retreat. This study and previous studies^5,10,30–33,35,37–41^ were simultaneously analyzed and incorporated to delineate a simplified feature with three stages: (1) early colonized community, (2) rapidly developed community, and (3) diversified and enriched community along with distance from the glacier.

To expand our focus on shift of polar benthic community structure, we conducted a mini-review and analyzed meta-data from literatures including the present study (Fig. 5). The result demonstrated that diversity and abundance of polar benthic organisms significantly vary with respect to ecological functioning groups. In other words, the functional diatom groups collectively contributed towards shifting the entire polar benthic community. The polar benthic community shift under the impacts of glacier retreat could be described in three stages. First, the new habitat exposed from retreating glacier and melting ice, then the diatoms melted out from sea ice during the warmer season. These diatoms formed a chain-like union of cells and quickly settled to the newly exposed substrates such as cobble and sediment. Life-forms and cell size are responses to various environmental condition^12^. The diatoms appeared to have a strategy to survive the fast-evolving harsh environment, which involved energy-efficient chain clustering^36^. Interestingly, sea ice pennate diatoms, such as *F. striatula*, dominated the intertidal zone, whereas centric diatoms, such as *Paralia* sp., dominated the subtidal zone.

Next, subsequently, microalgal dynamics would stabilize bottom habitats, providing refuge and potential diets to upper trophic organisms, such as meiofauna and/or macrofauna. A considerable number of copepods were observed that inhabited and ate the bushes of chain-form diatom, in the intertidal zone (Fig. 5). Limpets were the dominant macrofauna in the intertidal zone of the Marian Cove. In the subtidal zone, both meiofauna and macrofauna communities were characterized by dominance of opportunistic taxa (nematode^37^ and opportunistic ascidian^38^).

Finally, the diversified and enriched stage refers to the flourishing benthic communities through ecologically diverse diatom groups and abundant diatom (bloom) in the subtidal zone^5^, and higher diversity of meiofauna and macrofauna (Fig. 5). Ecological status in the outer intertidal zone, say old habitat, also represented stable community structure with predominance of tolerant species, viz., small motile naviculoid diatoms, to harsh conditions such as salinity fluctuation, wave action, etc.

The diatom communities inhabiting the subtidal zone were divided into two types. First, the dominance of epiphytic diatoms (> 30%) was featured in the subtidal sediments. Second, the chain-form diatom lived on macroalgal and/or macrofaunal colonies in the form of bushes, of which observation was documented by Ahn et al., 2016. Macrofaunal communities in the outer cove represented the matured colonization of macrofauna and/or megafauna, with dominant species including clams, sponges, ascidians, and echinoderms (author observation). During this stage, extensive algal mats of chain-forming diatoms attached to fauna are evidenced, representing the most mature colonization of the benthic polar community^5^. Thus, polar benthic communities are developed through the support of the benthic diatom, a rapid colonizer^35,39^, and promoted to diversified and enriched communities in the fast-evolving, harsh polar environment.

The current study is novel in that it investigated both intertidal type habitats and subtidal deep waters simultaneously for polar benthic diatoms. Interestingly, benthic diatom assemblages exhibited diverse ecological responses (with respect to occurrence, distribution, and diversity) to the given environmental settings associated with glacier retreat. First, epilithic diatoms primarily consisted of chain-forming species, which dominated the intertidal cove. Second, epibiotic diatoms on limpets show constant species composition regardless of sampling position (in both inner and outer cove). Finally, the species diversity of epiphytic diatoms varied greatly across locations, but tended to increase in older habitats (viz., habitats that were exposed earlier), confirming the occurrence of micro-floral community shift. Overall, benthic diatoms seem to represent appropriate and promising indicator taxa for monitoring and/or predicting the status of the sensitive polar benthic community and associated long-term changes under the current climate change regime.

## Conclusions

The present study highlighted the potential of using benthic diatoms as “indicator assemblages” on the effects of glacial retreat effects on polar benthic ecosystem. The occurrence, distribution, and signature taxa of benthic diatoms found in Marian Cove, West Antarctica, broadly demonstrated a series of ecological responses, from early colonization, to community development, diversified and enriched. Water depth and substrate type were identified as key factors that influenced species composition and/or abundance of polar benthic diatoms. The site-specific distributions of certain indicator species across the cove indicated the presence of taxa-dependent associations of benthic diatoms to oceanographic settings. The chain-forming strategy of *Fragilaria* spp. quickly adapted to newly exposed intertidal habitats, following recent glacial retreat. In comparison, *N*. cf. *perminuta,* dominating hard bottomed substrate, especially cobbles and limpet shells, in the intertidal zone. Several signature diatom taxa were identified as promising species for monitoring future changes to the benthic ecosystem of Marian Cove, West Antarctica, and, potentially, elsewhere in areas with retreating glaciers. Overall, the present study provides new insights on the responses and changes of marine ecosystem in sensitive polar regions under the current regime of global warming.

## Supplementary information

Supplementary Information.

## Data Availability

All data are available in the main text or the Supplementary information.
