# Supplementary material for: Shift in polar benthic community structure in a fast retreating glacial area of Marian Cove, West Antarctica
Source: Sci Rep. 2021 Jan 8;11:241. doi: 10.1038/s41598-020-80636-z (PMC7794547; doi:10.1038/s41598-020-80636-z)
Supplement: Supplementary file 1 — Supplementary Information. [file 41598_2020_80636_MOESM1_ESM.docx]

<Supporting Information>

**Shift in polar benthic community structure in a fast retreating
glacial area of Marian Cove, West Antarctica**

Hanna Bae, In-Young Ahn, Jinsoon Park, Sung Joon Song,
Junsung Noh, Hosang Kim, Jong Seong Khim

**Supplementary tables**

Table S1. Summary of statistics with specific purposes, data description, and software ············S3

Table S2. Data on the environmental variables measured at six intertidal locations (B1–B5 & B6 as a reference site), Marian Cove, West Antarctica ···················································S4

Table S3. Relative abundance (300 counts per sample) of benthic diatoms found at six intertidal locations (B1–B5 & B6 as a reference site), Marian Cove, West Antarctica, data given by type substrates of rock (film and mat forms), macroalgae, and limpet shell in 2018·········S5

Table S4. Relative abundance (300 counts per sample) of benthic diatoms on rocks at six intertidal locations (B1–B5 & B6 as a reference site), Marian Cove, West Antarctica, in 2019········S7

Table S5. Relative abundance (300 counts per sample) of benthic diatoms in subtidal sediments from four locations (M1–M4; at two depths of 10 and 30 m), Marian Cove, West Antarctica ·········································································································S8

Table S6. Diversity indices of benthic diatom communities at the intertidal and subtidal locations of Marian Cove, West Antarctica, by substrate type; d, species richness; *J’*, Pielou’s evenness; *H’*, Shannon wiener diversity····································································· S12

Table S7. IndVal analysis listing the indicator epilithic diatom species by clustered groups delineated for Marian Cove, West Antarctica. Given groups representing geographical setting, such as A: inner intertidal locations; B: outer intertidal locations; C: inner subtidal locations; D: outer subtidal locations·············································································S13

Table S8. Ecological type of benthic diatoms, by habitat preference, for those found at the intertidal and subtidal locations, Marian Cove, West Antarctica. Bibliographic information of references presented in Supplementary reference ··············································S14

**Supplementary figures**

Figure S1. Map showing (a) the study area, (b) sampling locations (n = 10) with site views (B1–B6) and observed algal biomass. (c–d) sampling design was presented including information of substrate. The base maps were created in Adobe Illustrator CS6 based on the map of Google Earth (https://earth.google.com/web/). ························································ S17

Figure S2. Light microscope photographs of centric diatoms in Marian Cove, West Antarctica. (**a–c**) *Actinocyclus* sp. 1; (**d–h**) *Melosira* sp. 1 (scale bar = 10 µm) ······························· S18

Figure S3. Light microscope photographs of centric diatoms in Marian Cove, West Antarctica. (**a**) *Paralia* sp. 1; (**b–g**) *Cocconeis* cf. *pinnata*; (**h**) *Cocconeis pinnata* var. *matsii*; (**i–j**) *Cocconeis* *melchiroides* ········································································· S19

Figure S4. Light microscope photographs of pennate diatoms in Marian Cove, West Antarctica. (**a–b**) *Achnanthes* sp. 3; (**c–f**) *Achnanthes brevipes* var. *intermedia*; (**g–h**) *Brandinia mosimanniae*; (**i–j**) *Fragilaria striatula*; (**k**) *Halamphora* sp. 1; (**l–m**) *Navicula* sp. 5 ······································································································ S20

Figure S5. Light microscope photographs of pennate diatoms in Marian Cove, West Antarctica. (**a**) *Fragilariopsis* *curta*; (**b**) *Fragilaria islandica* var. *adeliae*; (**c–e**) *Tabulariopsis cf. australis*; (**f–i**) *Navicula* cf. *perminuta*; (**j**) *Navicula* cf. *directa*; (**k**) *Nitzschia* sp. 1; (**l**) *Pinnularia* *australomicrostauron*; (**m**) *Planothidium delicatulum*; (**n**) *Synedropsis* *recta*; (**o–p**) *Navicula glaciei*; (**q–r**) *Pteroncola carlinii*; (**s–v**) *Pseudogomphonema kamtschaticum* ······································································································ S21

Figure S6. Scanning electron microscope (SEM) photographs of benthic diatoms in Marian Cove, West Antarctica. (**a**) *Melosira* sp. 1; (**b**) *Cocconeis* cf. *pinnata*; (**c–d**) *Achnanthes brevipes* var. *intermedia*; (**e**) *Navicula* sp*. 5*; (**f**) *Halamphora* sp. 1 (scale bar = 10 µm) ················ S22

Figure S7. Scanning electron microscope (SEM) photographs of benthic diatoms in Marian Cove, West Antarctica. (**a**) *Brandinia mosimanniae*; (**b**) *Fragilaira* sp. 1; (**c–f**) *Fragilaria islandica* var. *adeliae* (scale bar = 10 µm) ································································ S23

Figure S8. Scanning electron microscope (SEM) photographs of benthic diatoms in Marian Cove, West Antarctica. (**a**) *Fragilaria* cf. *striatula*; (**b**) *Licmophora gracilis*; (**c**) *Navicula* cf. *perminuta*; (**d–e**) *Navicula glaciei*; (**f**) *Pseudogomphonema kamtschaticum* (scale bar in **a–b**, and **d–e** = 10 µm, and in **f** = 2 µm) ······································································· S24

Figure S9. Illustration of the groups of benthic diatom assemblages in Marian Cove, West Antarctica, based on the NMDS (non-metric multidimensional scaling); five indicator species presented with corresponding indicator values and relative abundances (300 counts per sample) ····························································································· S25

**Supplementary references** ·················································································· S26

*** Corresponding authors.**

*E-mail addresses*: [jpark@kmou.ac.kr](mailto:jpark@kmou.ac.kr) (J. Park); [jskocean@snu.ac.kr](mailto:jskocean@snu.ac.kr) (J.S. Khim).

**Supplementary tables**

**Table S1.** Summary of statistics with specific purposes, data description, and software.

| **Method** | **Purpose** | **Remark** | **Software** |
| --- | --- | --- | --- |
| Cluster analysis | Characterize diatom groups | Abundance data square root transformed | PRIMER 6 |
| NMDS | Localize samples in two-dimensional space by site and by substrate | Abundance data square root transformed | PRIMER 6 |
| ANOSIM | Confirm whether clustered groups differ significantly | - | PRIMER 6 |
| SIMPER | Identify the species that contributed most to the dissimilarities within clustered groups | - | PRIMER 6 |
| IndVal | Identify indicator diatom species within each group following cluster analysis | IndVal*_ij_* = A*_ij_* _×_ B*_ij_* = 100  IndVal*_i_* = max[IndVal*_ij_*] | R studio |

A*_ij_*: specificity (i.e., the proportion of individuals of species *i* that were in class *j*),

B*_ij_*: fidelity (i.e., the proportion of sites in class *j* that included species *i*)

**Table S2.** Data on the environmental variables measured at six intertidal locations (B1–B5 & B6 as a reference site), Marian Cove, West Antarctica.

| **Environmental Variables** | | **2018** | | | | | |  | **2019** | | | | | |  | **Total** | | |
| --- | --- | --- | --- | --- | --- | --- | --- | --- | --- | --- | --- | --- | --- | --- | --- | --- | --- | --- |
|  |  | **B1** | **B2** | **B3** | **B4** | **B5** | **B6** |  | **B1** | **B2** | **B3** | **B4** | **B5** | **B6** |  | **Mean** | **Min** | **Max** |
| Temperature (°C) | | 1.1 | 2.1 | 1.3 | 1.5 | 1.9 | 2.8 |  | 1.3 | 1.8 | 6.1 | 5.1 | 2.3 | 2.7 |  | 2.4 | 1.1 | 6.1 |
| DO (mg L^−1^) | | 15.1 | 14.3 | 12.3 | 11.7 | 13.5 | 15.6 |  | 16.2 | 13.6 | 12.2 | 11.2 | 12.5 | 17.1 |  | 14.2 | 11.7 | 17.1 |
| Salinity (psu) | | 34.5 | 33.7 | 34.8 | 34.4 | 34.2 | 32.1 |  | 30.6 | 32.7 | 29.1 | 30.6 | 32.8 | 33.2 |  | 32.6 | 29.1 | 34.8 |
| pH | | 8.22 | 8.45 | 8.18 | 8.26 | 8.26 | 8.52 |  | 8.30 | 8.15 | 7.94 | 7.84 | 7.89 | 8.43 |  | 8.32 | 7.84 | 8.52 |
| Nutrients | SiO_2_ (μg L^-1^) | 0.47 | 0.38 | 0.39 | 0.43 | 0.38 | 0.50 |  | n.a.^*^ | n.a. | n.a. | n.a. | n.a. | n.a. |  | 0.42 | 0.38 | 0.50 |
|  | TP (μg L^-1^) | <0.01 | 0.1 | <0.01 | <0.01 | <0.01 | 0.1 |  | n.a. | n.a. | n.a. | n.a. | n.a. | n.a. |  | 0.1 | <0.01 | 0.1 |
|  | TN (μg L^-1^) | 0.51 | 0.78 | 0.53 | 0.61 | 0.52 | 1.84 |  | n.a. | n.a. | n.a. | n.a. | n.a. | n.a. |  | 0.8 | 0.5 | 1.8 |
| δ^13^C (‰) | POM | n.a. | −18.1 | n.a. | −20.3 | −24.5 | −24.9 |  | n.a. | −18.2 | n.a. | −18.4 | −23.8 | −24.1 |  | −21.5 | −24.9 | −18.1 |
|  | Diatom | n.a. | −16.0 | n.a. | −14.7 | −17.9 | −23.3 |  | n.a. | −16.6 | n.a. | −18.7 | −23.8 | −23.2 |  | −19.2 | −23.8 | −14.7 |
| δ^15^N (‰) | POM | n.a. | 4.4 | n.a. | 4.9 | 5.0 | 5.4 |  | n.a. | 7.2 | n.a. | 10.7 | 3.5 | 4.9 |  | 6.0 | 3.5 | 10.7 |
|  | Diatom | n.a. | 4.9 | n.a. | 6.3 | 5.5 | 7.4 |  | n.a. | 7.2 | n.a. | 6.3 | 7.2 | 7.5 |  | 6.4 | 4.9 | 7.5 |

^*^n.a.: not available

**Table S3.** Relative abundance (300 counts per sample) of benthic diatoms found at six intertidal locations (B1–B5 & B6 as a reference site), Marian Cove, West Antarctica, data given by type substrates of rock (film and mat forms), macroalgae, and limpet shell in 2018

| **Species** | **Total** | **Rock** | | | | | | | | |  | **Macroalgae** | | | | | |  | **Limpet shell** | | | |
| --- | --- | --- | --- | --- | --- | --- | --- | --- | --- | --- | --- | --- | --- | --- | --- | --- | --- | --- | --- | --- | --- | --- |
|  |  | **Film** | | | | | |  | **Mat** | |  |  |  |  |  |  |  |  |  |  |  |  |
|  |  | **B1** | **B2** | **B3** | **B4** | **B5** | **B6** |  | **B1** | **B2** |  | **B1** | **B2** | **B3** | **B4** | **B5** | **B6** |  | **B1** | **B4** | **B5** | **B6** |
| *Navicula* cf. *perminuta* | 2348 | 47 | 1 | 282 | 148 | 186 | 281 |  | 25 | 0 |  | 0 | 92 | 158 | 48 | 0 | 49 |  | 245 | 242 | 259 | 285 |
| *Fragilaria striatula* | 756 | 160 | 201 | 0 | 0 | 0 | 0 |  | 252 | 0 |  | 0 | 143 | 0 | 0 | 0 | 0 |  | 0 | 0 | 0 | 0 |
| *Pteroncola carlinii* | 290 | 0 | 0 | 0 | 0 | 5 | 0 |  | 4 | 0 |  | 0 | 0 | 2 | 135 | 0 | 105 |  | 26 | 13 | 0 | 0 |
| *Pseudogomphonema kamtschaticum* | 235 | 1 | 0 | 1 | 5 | 15 | 1 |  | 0 | 0 |  | 0 | 11 | 2 | 76 | 0 | 79 |  | 10 | 20 | 13 | 1 |
| *Navicula glaciei* | 185 | 5 | 29 | 4 | 0 | 24 | 9 |  | 9 | 22 |  | 2 | 33 | 39 | 0 | 0 | 0 |  | 6 | 3 | 0 | 0 |
| *Achnanthes brevipes* var. *intermedia* | 43 | 0 | 0 | 0 | 0 | 0 | 0 |  | 0 | 25 |  | 0 | 17 | 1 | 0 | 0 | 0 |  | 0 | 0 | 0 | 0 |
| *Fragilaria* cf. *striatula* | 112 | 6 | 0 | 0 | 0 | 4 | 0 |  | 0 | 102 |  | 0 | 0 | 0 | 0 | 0 | 0 |  | 0 | 0 | 0 | 0 |
| *Fragilaria islandica* var. *adeliae* | 104 | 0 | 0 | 0 | 0 | 0 | 0 |  | 2 | 88 |  | 0 | 2 | 0 | 0 | 0 | 0 |  | 0 | 0 | 12 | 0 |
| *Cocconeis pinnata* var. *matsii* | 83 | 0 | 0 | 0 | 0 | 0 | 0 |  | 0 | 0 |  | 0 | 0 | 83 | 0 | 0 | 0 |  | 0 | 0 | 0 | 0 |
| *Achnanthes* sp. 1 | 63 | 0 | 0 | 0 | 0 | 63 | 0 |  | 0 | 0 |  | 0 | 0 | 0 | 0 | 0 | 0 |  | 0 | 0 | 0 | 0 |
| *Achnanthes* cf. *bongrainii* | 55 | 8 | 47 | 0 | 0 | 0 | 0 |  | 0 | 0 |  | 0 | 0 | 0 | 0 | 0 | 0 |  | 0 | 0 | 0 | 0 |
| *Parlibellus crucicula* | 50 | 48 | 0 | 0 | 0 | 0 | 0 |  | 2 | 0 |  | 0 | 0 | 0 | 0 | 0 | 0 |  | 0 | 0 | 0 | 0 |
| *Licmophora antarctica* | 48 | 0 | 1 | 5 | 0 | 0 | 0 |  | 0 | 33 |  | 0 | 1 | 0 | 0 | 0 | 8 |  | 0 | 0 | 0 | 0 |
| *Licmophora* cf. *gracilis* | 37 | 0 | 0 | 0 | 0 | 0 | 0 |  | 0 | 0 |  | 0 | 0 | 0 | 0 | 0 | 30 |  | 7 | 0 | 0 | 0 |
| *Licmophora gracilis* | 33 | 0 | 1 | 0 | 0 | 0 | 0 |  | 0 | 4 |  | 0 | 0 | 0 | 28 | 0 | 0 |  | 0 | 0 | 0 | 0 |
| *Pinnularia* sp. 1 | 25 | 18 | 0 | 0 | 0 | 0 | 0 |  | 0 | 0 |  | 0 | 0 | 0 | 0 | 0 | 0 |  | 0 | 7 | 0 | 0 |
| *Cocconeis* cf. *pinnata* | 23 | 0 | 0 | 0 | 15 | 0 | 0 |  | 0 | 0 |  | 0 | 0 | 0 | 8 | 0 | 0 |  | 0 | 0 | 0 | 0 |
| *Brandinia mosimanniae* | 19 | 5 | 8 | 0 | 0 | 2 | 0 |  | 0 | 0 |  | 0 | 0 | 0 | 0 | 0 | 0 |  | 4 | 0 | 0 | 0 |
| cf. *Gomphonemopsis obscura* | 18 | 0 | 0 | 4 | 0 | 0 | 0 |  | 0 | 0 |  | 0 | 0 | 14 | 0 | 0 | 0 |  | 0 | 0 | 0 | 0 |
| *Cocconeis melchioroides* | 18 | 0 | 0 | 0 | 0 | 0 | 2 |  | 0 | 0 |  | 0 | 0 | 0 | 0 | 0 | 16 |  | 0 | 0 | 0 | 0 |
| *Tabulariopsis cf. australis* | 17 | 0 | 0 | 0 | 0 | 0 | 0 |  | 0 | 17 |  | 0 | 0 | 0 | 0 | 0 | 0 |  | 0 | 0 | 0 | 0 |
| *Navicula* sp*.* 5 | 12 | 0 | 0 | 0 | 0 | 0 | 0 |  | 0 | 9 |  | 0 | 0 | 0 | 0 | 0 | 0 |  | 1 | 0 | 0 | 2 |
| *Paralia* sp. 1 | 11 | 0 | 0 | 0 | 0 | 0 | 0 |  | 0 | 0 |  | 0 | 0 | 0 | 0 | 0 | 0 |  | 0 | 11 | 0 | 0 |

**Table S3.** (continued)

| **Species** | **Total** | **Rock** | | | | | | | | |  | **Macroalgae** | | | | | |  | **Limpet shell** | | | |
| --- | --- | --- | --- | --- | --- | --- | --- | --- | --- | --- | --- | --- | --- | --- | --- | --- | --- | --- | --- | --- | --- | --- |
|  |  | **Film** | | | | | |  | **Mat** | |  |  |  |  |  |  |  |  |  |  |  |  |
|  |  | **B1** | **B2** | **B3** | **B4** | **B5** | **B6** |  | **B1** | **B2** |  | **B1** | **B2** | **B3** | **B4** | **B5** | **B6** |  | **B1** | **B4** | **B5** | **B6** |
| *Thalassiosira* sp. 3 | 11 | 0 | 0 | 0 | 0 | 0 | 0 |  | 0 | 0 |  | 0 | 0 | 0 | 0 | 0 | 0 |  | 0 | 0 | 0 | 11 |
| *Cocconeis costata* var. *antarctica* | 10 | 0 | 0 | 0 | 9 | 0 | 1 |  | 0 | 0 |  | 0 | 0 | 0 | 0 | 0 | 0 |  | 0 | 0 | 0 | 0 |
| *Odontella litigosa* | 10 | 0 | 10 | 0 | 0 | 0 | 0 |  | 0 | 0 |  | 0 | 0 | 0 | 0 | 0 | 0 |  | 0 | 0 | 0 | 0 |
| *Pseudogomphonema* sp. 1 | 10 | 0 | 0 | 0 | 0 | 0 | 0 |  | 0 | 0 |  | 0 | 0 | 0 | 0 | 0 | 10 |  | 0 | 0 | 0 | 0 |
| *Melosira* sp. 1 | 9 | 0 | 0 | 0 | 0 | 0 | 0 |  | 0 | 0 |  | 0 | 0 | 0 | 2 | 0 | 0 |  | 0 | 0 | 7 | 0 |
| *Navicula directa* | 9 | 0 | 0 | 0 | 0 | 0 | 0 |  | 2 | 0 |  | 0 | 0 | 0 | 3 | 0 | 0 |  | 0 | 4 | 0 | 0 |
| *Cyclotella* sp. 1 | 7 | 0 | 0 | 0 | 0 | 0 | 2 |  | 0 | 0 |  | 0 | 0 | 0 | 0 | 0 | 0 |  | 0 | 0 | 5 | 0 |
| *Rhoicosphenia* sp. 2 | 4 | 0 | 0 | 4 | 0 | 0 | 0 |  | 0 | 0 |  | 0 | 0 | 0 | 0 | 0 | 0 |  | 0 | 0 | 0 | 0 |
| *Nitzschia cf*. *wilmotteana* | 3 | 0 | 0 | 0 | 0 | 0 | 0 |  | 0 | 0 |  | 0 | 0 | 0 | 0 | 0 | 0 |  | 0 | 0 | 3 | 0 |
| *Planothidium* cf. *delicatulum* | 3 | 2 | 0 | 0 | 0 | 1 | 0 |  | 0 | 0 |  | 0 | 0 | 0 | 0 | 0 | 0 |  | 0 | 0 | 0 | 0 |
| *Licmophora luxuriosa* | 3 | 0 | 0 | 0 | 0 | 0 | 0 |  | 3 | 0 |  | 0 | 0 | 0 | 0 | 0 | 0 |  | 0 | 0 | 0 | 0 |
| *Navicula cf*. *cancellata* | 3 | 0 | 0 | 0 | 0 | 0 | 3 |  | 0 | 0 |  | 0 | 0 | 0 | 0 | 0 | 0 |  | 0 | 0 | 0 | 0 |
| *Achnanthes* sp. 2 | 2 | 0 | 2 | 0 | 0 | 0 | 0 |  | 0 | 0 |  | 0 | 0 | 0 | 0 | 0 | 0 |  | 0 | 0 | 0 | 0 |
| *Achnanthes* sp. 3 | 2 | 0 | 0 | 0 | 0 | 0 | 0 |  | 0 | 0 |  | 0 | 0 | 1 | 0 | 0 | 0 |  | 0 | 0 | 1 | 0 |
| *Navicula* sp. 1 | 2 | 0 | 0 | 0 | 0 | 0 | 0 |  | 0 | 0 |  | 0 | 0 | 0 | 0 | 0 | 2 |  | 0 | 0 | 0 | 0 |
| *Nitzschia* cf. *homburgiensis* | 1 | 0 | 0 | 0 | 0 | 0 | 0 |  | 0 | 0 |  | 0 | 0 | 0 | 0 | 0 | 0 |  | 0 | 0 | 0 | 1 |
| *Planothidium* sp. 1 | 1 | 0 | 0 | 0 | 0 | 0 | 0 |  | 1 | 0 |  | 0 | 0 | 0 | 0 | 0 | 0 |  | 0 | 0 | 0 | 0 |
| *Cocconeis costata* | 1 | 0 | 0 | 0 | 0 | 0 | 0 |  | 0 | 0 |  | 0 | 1 | 0 | 0 | 0 | 0 |  | 0 | 0 | 0 | 0 |
| *Cocconeis pottercovei* | 1 | 0 | 0 | 0 | 0 | 0 | 1 |  | 0 | 0 |  | 0 | 0 | 0 | 0 | 0 | 0 |  | 0 | 0 | 0 | 0 |
| *Navicula* sp. 3 | 1 | 0 | 0 | 0 | 0 | 0 | 0 |  | 0 | 0 |  | 0 | 0 | 0 | 0 | 0 | 0 |  | 1 | 0 | 0 | 0 |
| *Rhoicosphenia* sp. 1 | 1 | 0 | 0 | 0 | 0 | 0 | 0 |  | 0 | 0 |  | 0 | 0 | 0 | 0 | 0 | 1 |  | 0 | 0 | 0 | 0 |

**Table S4.** Relative abundance (300 counts per sample) of benthic diatoms on rocks at six intertidal locations (B1–B5 & B6 as a reference site), Marian Cove, West Antarctica, in 2019.

| **Species** | **Total** |  | **Station** | | | | | |
| --- | --- | --- | --- | --- | --- | --- | --- | --- |
|  |  |  | **B1** | **B2** | **B3** | **B4** | **B5** | **B6** |
| *Navicula* cf. *perminuta* | 1187 |  | 49 | 187 | 282 | 256 | 165 | 248 |
| *Fragilaria striatula* | 195 |  | 157 | 36 | 0 | 0 | 2 | 0 |
| *Achnanthes brevipes* var. *intermedia* | 166 |  | 3 | 11 | 3 | 34 | 113 | 2 |
| *Navicula glaciei* | 95 |  | 76 | 12 | 2 | 4 | 1 | 0 |
| *Synedropsis recta* | 41 |  | 1 | 19 | 9 | 2 | 7 | 3 |
| *Pseudogomphonema kamtschaticum* | 34 |  | 6 | 11 | 0 | 2 | 3 | 12 |
| *Pteroncola carlinii* | 21 |  | 0 | 0 | 0 | 0 | 3 | 18 |
| *Planothidium* cf. *delicatulum* | 18 |  |  | 13 | 2 |  |  | 3 |
| *Brandinia mosimanniae* | 12 |  | 5 | 5 | 0 | 0 | 2 | 0 |
| *Cocconeis* cf. *pinnata* | 9 |  | 0 | 2 | 0 | 1 | 1 | 5 |
| *Licmophora gracilis* | 6 |  | 0 | 0 | 0 | 0 | 1 | 5 |
| *Nitzschia pellucida* | 6 |  | 0 | 4 | 1 | 0 | 0 | 1 |
| *Nitzschia* sp. 1 | 3 |  | 0 | 0 | 0 | 0 | 0 | 3 |
| *Fragilaria* cf. *striatula* | 3 |  | 3 | 0 | 0 | 0 | 0 | 0 |
| *Fragilariopsis curta* | 2 |  | 0 | 0 | 0 | 0 | 2 | 0 |
| *Synedropsis laevis* | 1 |  | 0 | 0 | 1 | 0 | 0 | 0 |
| *Cocconeis costata* var. *antarctica* | 1 |  | 0 | 0 | 0 | 1 | 0 | 0 |

**Table S5.** Relative abundance (300 counts per sample) of benthic diatoms in subtidal sediments from four locations (M1–M4; at two depths of 10 and 30 m), Marian Cove, West Antarctica.

| **Species** | **Total** | **M1** | |  | **M2** | |  | **M3** | |  | **M4** | |
| --- | --- | --- | --- | --- | --- | --- | --- | --- | --- | --- | --- | --- |
|  |  | **10 m** | **30 m** |  | **10 m** | **30 m** |  | **10 m** | **30 m** |  | **10 m** | **30 m** |
| *Navicula* cf. *perminuta* | 400 | 29 | 75 |  | 60 | 46 |  | 77 | 32 |  | 40 | 41 |
| *Navicula glaciei* | 381 | 175 | 55 |  | 73 | 38 |  | 17 | 13 |  | 5 | 5 |
| *Pseudogomphonema kamtschaticum* | 202 | 11 | 4 |  | 18 | 11 |  | 37 | 30 |  | 71 | 20 |
| *Navicula directa* | 161 | 11 | 36 |  | 12 | 24 |  | 17 | 40 |  | 4 | 17 |
| *Cocconeis* cf. *pinnata* | 145 | 6 | 3 |  | 14 | 0 |  | 11 | 5 |  | 51 | 55 |
| *Achnanthes brevipes* var. *intermedia* | 90 | 0 | 14 |  | 12 | 28 |  | 2 | 10 |  | 12 | 12 |
| *Synedropsis recta* | 74 | 0 | 0 |  | 0 | 0 |  | 25 | 26 |  | 12 | 11 |
| *Cocconeis pottercovei* | 69 | 1 | 0 |  | 2 | 4 |  | 13 | 10 |  | 31 | 8 |
| *Fragilaria striatula* | 66 | 15 | 19 |  | 16 | 6 |  | 5 | 2 |  | 2 | 1 |
| *Nitzschia* sp. 1 | 58 | 1 | 7 |  | 0 | 2 |  | 34 | 13 |  | 1 | 0 |
| *Navicula* cf. *cancellata* | 53 | 21 | 2 |  | 7 | 10 |  | 3 | 3 |  | 2 | 5 |
| *Planothidium* cf. *delicatulum* | 43 | 5 | 0 |  | 9 | 17 |  | 3 | 1 |  | 1 | 7 |
| *Cocconeis* cf. *scutellum* | 42 | 0 | 0 |  | 0 | 0 |  | 2 | 6 |  | 7 | 27 |
| *Cocconeis pinnata* var. *matsii* | 35 | 0 | 0 |  | 0 | 0 |  | 4 | 8 |  | 23 | 0 |
| *Fragilariopsis curta* | 35 | 0 | 16 |  | 7 | 0 |  | 0 | 6 |  | 1 | 5 |
| *Synedropsis laevis* | 30 | 7 | 2 |  | 14 | 5 |  | 0 | 0 |  | 2 | 0 |
| *Cocconeis costata* var. *antarctica* | 28 | 0 | 5 |  | 3 | 20 |  | 0 | 0 |  | 0 | 0 |
| *Pinnularia australomicrostauron* | 25 | 0 | 0 |  | 0 | 25 |  | 0 | 0 |  | 0 | 0 |
| *Pseudogomphonema* sp. 2 | 24 | 0 | 0 |  | 0 | 0 |  | 6 | 6 |  | 9 | 3 |
| *Actinocyclus* sp. 1 | 23 | 0 | 0 |  | 0 | 0 |  | 2 | 9 |  | 2 | 10 |
| *Paralia* sp. 1 | 23 | 0 | 0 |  | 0 | 1 |  | 0 | 4 |  | 2 | 16 |
| *Nitzschia* sp. 3 | 20 | 1 | 0 |  | 16 | 0 |  | 0 | 2 |  | 0 | 1 |
| *Thalassionema* cf. *nitzschioides* | 20 | 0 | 0 |  | 0 | 0 |  | 1 | 18 |  | 0 | 1 |
| *Licmophora luxuriosa* | 18 | 0 | 7 |  | 0 | 11 |  | 0 | 0 |  | 0 | 0 |

**Table S5.** (continued)

| **Species** | **Total** | **M1** | |  | **M2** | |  | **M3** | |  | **M4** | |
| --- | --- | --- | --- | --- | --- | --- | --- | --- | --- | --- | --- | --- |
|  |  | **10 m** | **30 m** |  | **10 m** | **30 m** |  | **10 m** | **30 m** |  | **10 m** | **30 m** |
| *Nitzschia* cf. *homburgiensis* | 18 | 0 | 4 |  | 11 | 2 |  | 0 | 0 |  | 1 | 0 |
| *Haslea* sp. 1 | 17 | 0 | 14 |  | 3 | 0 |  | 0 | 0 |  | 0 | 0 |
| *Navicula* sp. 2 | 17 | 0 | 0 |  | 0 | 0 |  | 8 | 5 |  | 0 | 4 |
| *Pinnularia* sp. 2 | 15 | 0 | 6 |  | 0 | 8 |  | 0 | 0 |  | 0 | 1 |
| *Synedra* cf. *kerguelensis* | 13 | 0 | 0 |  | 0 | 13 |  | 0 | 0 |  | 0 | 0 |
| *Pseudogomphonema* sp. 1 | 12 | 0 | 0 |  | 0 | 0 |  | 0 | 2 |  | 10 | 0 |
| *Cyclotella* sp. 1 | 11 | 7 | 0 |  | 3 | 1 |  | 0 | 0 |  | 0 | 0 |
| *Nitzschia* sp. 4 | 11 | 0 | 0 |  | 0 | 0 |  | 3 | 1 |  | 0 | 7 |
| *Odontella litigosa* | 11 | 0 | 0 |  | 0 | 0 |  | 0 | 1 |  | 0 | 10 |
| *Tabularia tabulata* | 11 | 0 | 0 |  | 0 | 0 |  | 2 | 1 |  | 0 | 8 |
| *Thalassiosira* sp. 3 | 11 | 0 | 8 |  | 0 | 3 |  | 0 | 0 |  | 0 | 0 |
| *Achnanthes* cf. *bongrainii* | 9 | 5 | 2 |  | 0 | 0 |  | 2 | 0 |  | 0 | 0 |
| *Cocconeis melchioroides* | 9 | 0 | 0 |  | 0 | 0 |  | 0 | 8 |  | 0 | 1 |
| *Navicula* sp. 4 | 9 | 0 | 0 |  | 0 | 0 |  | 4 | 5 |  | 0 | 0 |
| *Fallacia marnierii* | 8 | 0 | 0 |  | 0 | 0 |  | 3 | 3 |  | 0 | 2 |
| *Nitzschia* cf. *gracilis* | 8 | 0 | 0 |  | 5 | 3 |  | 0 | 0 |  | 0 | 0 |
| *Thalassiosira* sp. 1 | 8 | 1 | 6 |  | 0 | 0 |  | 0 | 0 |  | 1 | 0 |
| *Nitzschia* cf. *wilmotteana* | 7 | 0 | 6 |  | 0 | 0 |  | 0 | 0 |  | 1 | 0 |
| *Pleurosigma* cf. *obscurum* | 7 | 0 | 0 |  | 0 | 0 |  | 4 | 3 |  | 0 | 0 |
| *Amphora coffeaeformis* | 6 | 0 | 0 |  | 4 | 0 |  | 0 | 0 |  | 1 | 1 |
| *Cocconeis* cf. *imperatrix* | 6 | 0 | 0 |  | 0 | 0 |  | 0 | 0 |  | 0 | 6 |
| *Fragilariopsis separanda* | 6 | 0 | 0 |  | 0 | 0 |  | 0 | 5 |  | 0 | 1 |
| *Fragilariopsis* sp. 1 | 6 | 1 | 0 |  | 5 | 0 |  | 0 | 0 |  | 0 | 0 |
| *Melosira* sp. 1 | 6 | 0 | 0 |  | 0 | 0 |  | 0 | 3 |  | 0 | 3 |
| *Pteroncola carlinii* | 6 | 0 | 0 |  | 0 | 3 |  | 0 | 0 |  | 3 | 0 |

**Table S5.** (continued)

| **Species** | **Total** | **M1** | |  | **M2** | |  | **M3** | |  | **M4** | |
| --- | --- | --- | --- | --- | --- | --- | --- | --- | --- | --- | --- | --- |
|  |  | **10 m** | **30 m** |  | **10 m** | **30 m** |  | **10 m** | **30 m** |  | **10 m** | **30 m** |
| *Rhoicosphenia* sp. 1 | 6 | 0 | 1 |  | 0 | 5 |  | 0 | 0 |  | 0 | 0 |
| *Cocconeis schuetti* | 5 | 0 | 0 |  | 0 | 0 |  | 3 | 2 |  | 0 | 0 |
| *Cocconeis* sp. 1 | 5 | 2 | 1 |  | 0 | 2 |  | 0 | 0 |  | 0 | 0 |
| *Fragilaria* cf. *striatula* | 5 | 0 | 0 |  | 1 | 1 |  | 1 | 1 |  | 0 | 1 |
| *Fragilaria islandica* var. *adeliae* | 5 | 0 | 0 |  | 0 | 0 |  | 1 | 0 |  | 0 | 4 |
| *Pleurosigma* sp. 1 | 5 | 0 | 4 |  | 0 | 1 |  | 0 | 0 |  | 0 | 0 |
| *Achnanthes* sp. 1 | 4 | 0 | 0 |  | 3 | 1 |  | 0 | 0 |  | 0 | 0 |
| *Navicula* sp. 5 | 4 | 0 | 0 |  | 0 | 0 |  | 0 | 2 |  | 0 | 2 |
| *Amphora marina* | 4 | 0 | 0 |  | 0 | 0 |  | 0 | 2 |  | 0 | 2 |
| *Thalassiosira* cf. *punctigera* | 4 | 0 | 0 |  | 1 | 3 |  | 0 | 0 |  | 0 | 0 |
| *Corethron* sp. 1 | 3 | 0 | 0 |  | 0 | 0 |  | 1 | 2 |  | 0 | 0 |
| *Entomoneis* sp. 1 | 3 | 0 | 0 |  | 0 | 0 |  | 1 | 2 |  | 0 | 0 |
| *Tabulariopsis cf. australis* | 3 | 1 | 0 |  | 1 | 0 |  | 1 | 0 |  | 0 | 0 |
| *Gyrosigma fasciola* | 3 | 0 | 0 |  | 0 | 0 |  | 3 | 0 |  | 0 | 0 |
| *Navicula* sp. 3 | 3 | 0 | 0 |  | 0 | 0 |  | 0 | 3 |  | 0 | 0 |
| *Parlibellus crucicula* | 3 | 0 | 0 |  | 0 | 0 |  | 0 | 2 |  | 1 | 0 |
| *Achnanthes* sp. 3 | 2 | 0 | 0 |  | 0 | 0 |  | 1 | 0 |  | 1 | 0 |
| *Amphora* cf. *proteus* | 2 | 0 | 2 |  | 0 | 0 |  | 0 | 0 |  | 0 | 0 |
| *Cocconeis imperatrix* | 2 | 0 | 0 |  | 0 | 0 |  | 0 | 0 |  | 2 | 0 |
| *Nitzschia* sp. 2 | 2 | 0 | 0 |  | 0 | 1 |  | 1 | 0 |  | 0 | 0 |
| *Rhoicosphenia genuflexa* | 2 | 0 | 0 |  | 0 | 0 |  | 2 | 0 |  | 0 | 0 |
| *Thalassiosira* sp. 2 | 2 | 0 | 0 |  | 0 | 2 |  | 0 | 0 |  | 0 | 0 |
| *Actinocyclus actinochilus* | 1 | 0 | 0 |  | 0 | 0 |  | 0 | 1 |  | 0 | 0 |
| *Actinocyclus curvatulus* | 1 | 0 | 0 |  | 0 | 0 |  | 0 | 0 |  | 1 | 0 |
| *Cocconeis californica* | 1 | 0 | 0 |  | 0 | 0 |  | 0 | 1 |  | 0 | 0 |

**Table S5.** (continued)

| **Species** | **Total** | **M1** | |  | **M2** | |  | **M3** | |  | **M4** | |
| --- | --- | --- | --- | --- | --- | --- | --- | --- | --- | --- | --- | --- |
|  |  | **10 m** | **30 m** |  | **10 m** | **30 m** |  | **10 m** | **30 m** |  | **10 m** | **30 m** |
| *Diploneis* sp. 1 | 1 | 0 | 0 |  | 0 | 1 |  | 0 | 0 |  | 0 | 0 |
| *Entomoneis* sp. 2 | 1 | 0 | 0 |  | 0 | 1 |  | 0 | 0 |  | 0 | 0 |
| *Eucampia antarctica* | 1 | 0 | 0 |  | 0 | 0 |  | 0 | 0 |  | 0 | 1 |
| *Halamphora* sp. 1 | 1 | 0 | 0 |  | 0 | 1 |  | 0 | 0 |  | 0 | 0 |
| *Licmophora gracilis* | 1 | 0 | 0 |  | 0 | 0 |  | 0 | 1 |  | 0 | 0 |
| *Luticola* sp. 1 | 1 | 0 | 1 |  | 0 | 0 |  | 0 | 0 |  | 0 | 0 |
| *Rhabdonema arcuatum* | 1 | 0 | 0 |  | 0 | 0 |  | 0 | 0 |  | 0 | 1 |

**Table S6.** Diversity indices of benthic diatom communities at the intertidal and subtidal locations of Marian Cove, West Antarctica, by substrate type; d, species richness; *J’*, Pielou’s evenness; *H’*, Shannon wiener diversity.

| **Habitat (year)** | **Location** | | **Type** | **d** | ***J'*** | ***H'*** |
| --- | --- | --- | --- | --- | --- | --- |
| Intertidal (2018) | B1 | | Epilithic (mat) | 1.4 | 0.3 | 0.7 |
|  |  |  | Epilithic (film) | 1.4 | 0.6 | 1.4 |
|  |  |  | Epiphytic | 0 | - | 0 |
|  |  |  | Epizoic | 1.2 | 0.4 | 0.8 |
|  | B2 | | Epilithic (mat) | 1.2 | 0.8 | 1.7 |
|  |  |  | Epilithic (film) | 1.2 | 0.5 | 1.0 |
|  |  | | Epiphytic | 1.2 | 0.6 | 1.3 |
|  | B3 | | Epilithic (film) | 0.9 | 0.2 | 0.3 |
|  |  | | Epiphytic | 1.2 | 0.6 | 1.2 |
|  | B4 | | Epilithic (film) | 0.7 | 0.6 | 1.0 |
|  |  |  | Epiphytic | 1.1 | 0.7 | 1.4 |
|  |  | | Epizoic | 1.1 | 0.4 | 1.0 |
|  | B5 | | Epilithic (film) | 1.2 | 0.6 | 1.2 |
|  |  | | Epizoic | 1.1 | 0.3 | 0.6 |
|  | BR | | Epilithic (film) | 1.2 | 0.2 | 0.3 |
|  |  |  | Epiphytic | 1.4 | 0.8 | 1.7 |
|  |  | | Epizoic | 0.7 | 0.2 | 0.2 |
| Intertidal (2019) | B1 | | Epilithic (film) | 1.2 | 0.6 | 1.2 |
|  | B2 | | Epilithic (film) | 1.6 | 0.6 | 1.4 |
|  | B3 | | Epilithic (film) | 1.1 | 0.2 | 0.3 |
|  | B4 | | Epilithic (film) | 1.1 | 0.3 | 0.5 |
|  | B5 | | Epilithic (film) | 1.8 | 0.4 | 1.0 |
|  | B6 | | Epilithic (film) | 1.6 | 0.3 | 0.8 |
| Subtidal (2018) | M1 | (10 m)* | Sediment | 3.0 | 0.6 | 1.7 |
|  |  | (30 m) | Sediment | 4.2 | 0.8 | 2.5 |
|  | M2 | (10 m) | Sediment | 4.0 | 0.8 | 2.6 |
|  |  | (30 m) | Sediment | 5.1 | 0.8 | 2.9 |
|  | M3 | (10 m) | Sediment | 5.8 | 0.8 | 2.7 |
|  |  | (30 m) | Sediment | 6.7 | 0.9 | 3.1 |
|  | M4 | (10 m) | Sediment | 4.9 | 0.7 | 2.5 |
|  |  | (30 m) | Sediment | 6.7 | 0.8 | 3.0 |

*Water depth

**Table S7.** IndVal analysis listing the indicator epilithic diatom species by clustered groups delineated for Marian Cove, West Antarctica. Given groups representing geographical setting, such as A: inner intertidal locations; B: outer intertidal locations; C: inner subtidal locations; D: outer subtidal locations.

| **Indicator species** | **Group** | | | |
| --- | --- | --- | --- | --- |
|  | **A** | **B** | **C** | **D** |
| *Fragilaria striatula* | 0.91^***^ | 0.00 | 0.07 | 0.01 |
| *Navicula* cf. *perminuta* | 0.12 | 0.59^***^ | 0.15 | 0.14 |
| *Navicula glaciei* | 0.23 | 0.03 | 0.65^*^ | 0.08 |
| *Navicula directa* | 0.00 | 0.00 | 0.51^*^ | 0.47 |
| *Cocconeis* cf. *pinnata* | 0.00 | 0.02 | 0.11 | 0.80^**^ |
| *Pseudogomphonema kamtschaticum* | 0.03 | 0.21 | 0.16 | 0.57^*^ |

^*^*p* < 0.05, ^**^*p* < 0.01, ^***^p < 0.001

**Table S8.** Ecological type of benthic diatoms, by habitat preference, for those found at the intertidal and subtidal locations, Marian Cove, West Antarctica. Bibliographic information of references presented in Supplementary reference.

| **Species** | **Ecological type^*^** | | | **Reference** |
| --- | --- | --- | --- | --- |
|  | **m** | **eu** | **f** |  |
| *Achnanthes brevipes* var. *intermedia* | v | v | v | Cremer et al., 2003 |
| *Achnanthes* sp. 1 | v |  |  | Round et al., 1990 |
| *Achnanthes* sp. 2 | v |  |  | Round et al., 1990 |
| *Achnanthes* sp. 3 | v |  |  | Round et al., 1990 |
| *Achnanthes* sp. 4 | v |  |  | Round et al., 1990 |
| *Actinocyclus actinochilus* | v |  |  | Cremer et al., 2003 |
| *Actinocyclus curvatulus* | v |  |  | Hasle and Syvertsen, 1996 |
| *Actinocyclus* sp. 1 | v |  |  | Round et al., 1990 |
| *Amphora* cf. *proteus* | v |  |  | Witkowski et al., 2000 |
| *Amphora coffeaeformis* | v | v |  | Cremer et al., 2003 |
| *Amphora marina* | v |  |  | Al-Handal and Wulff, 2008a |
| *Brandinia mosimanniae* | v |  |  | Fernandes et al., 2007 |
| cf. *Gomphonemopsis obscura* | v |  |  | Witkowski et al., 2000 |
| *Cocconeis californica* | v |  |  | Al-Handal and Wulff, 2008a |
| *Cocconeis* cf. *imperatrix* | v |  |  | Al-Handal and Wulff, 2008a |
| *Cocconeis* cf. *scutellum* | v | v |  | Al-Handal and Wulff, 2008a |
| *Cocconeis costata* | v |  |  | Al-Handal and Wulff, 2008a |
| *Cocconeis costata* var. *antarctica* | v |  |  | Al-Handal and Wulff, 2008a |
| *Cocconeis imperatrix* | v |  |  | Al-Handal and Wulff, 2008a |
| *Cocconeis melchioroides* | v |  |  | Al-Handal and Wulff, 2008b |
| *Cocconeis* cf. *pinnata* | v |  |  | Al-Handal and Wulff, 2008a |
| *Cocconeis pinnata* var. *matsii* | v |  |  | Al-Handal et al., 2010 |
| *Cocconeis pottercovei* | v |  |  | Al-Handal et al., 2010 |
| *Cocconeis schuettii* | v |  |  | Al-Handal and Wulff, 2008a |
| *Cocconeis* sp. 1 | v |  |  | Al-Handal and Wulff, 2008a |
| *Corethron* sp. 1 | v |  |  | Round et al., 1990 |
| *Cyclotella* sp. 1 | v |  |  | Expert judge |
| *Diploneis* sp. 1 | v |  |  | Expert judge |
| *Entomoneis* sp. 1 | v |  |  | Expert judge |
| *Entomoneis* sp. 2 | v |  |  | Expert judge |
| *Eucampia antarctica* | v |  |  | Al-Handal and Wulff, 2008a |
| *Fallacia marnierii* | v |  |  | Cremer et al., 2003 |
| *Fragilaria* cf. *striatula* | v |  |  | Cremer et al., 2003 |
| *Fragilaria islandica* var. *adeliae* | v |  |  | Cremer et al., 2003 |
| *Tabulariopsis cf. australis* | v |  |  | Expert judge |
| *Fragilaria striatula* | v |  |  | Al-Handal and Wulff, 2008b |
| *Fragilariopsis curta* | v |  |  | Cremer et al., 2003 |
| *Fragilariopsis separanda* | v |  |  | Cremer et al., 2003 |
| *Fragilariopsis* sp. 1 | v |  |  | Cremer et al., 2003 |
| *Gyrosigma fasciola* | v | v |  | Al-Handal and Wulff, 2008a |
| *Halamphora* sp. 1 | v |  |  | Cremer et al., 2003 |

**Table S8.** (Continued)

| **Species** | **Ecological type^*^** | | | **Reference** |
| --- | --- | --- | --- | --- |
|  | **m** | **eu** | **f** |  |
| *Haslea* sp. 1 | v |  |  | Al-Handal and Wulff, 2008a |
| *Licmophora antarctica* | v |  |  | Fernandes et al., 2014 |
| *Licmophora* cf. *gracilis* | v |  |  | Round et al., 1990 |
| *Licmophora gracilis* | v |  |  | Cremer et al., 2003 |
| *Licmophora luxuriosa* | v |  |  | Al-Handal and Wulff, 2008a |
| *Luticola* sp. 1 |  |  | v | Round et al., 1990 |
| *Melosira* sp. 1 | v |  |  | Al-Handal and Wulff, 2008a |
| *Navicula* cf. *cancellata* | v |  |  | Expert judge |
| *Navicula* cf. *perminuta* | v | v | v | Al-Handal and Wulff, 2008a, Cremer et al., 2003 |
| *Navicula directa* | v |  |  | Expert judge |
| *Navicula* *glaciei* | v |  |  | Whitaker and Richardson, 1980; Kang et al., 1999 |
| *Navicula sp. 1* | v |  |  | Expert judge |
| *Navicula sp. 2* | v |  |  | Expert judge |
| *Navicula sp. 3* | v |  |  | Expert judge |
| *Navicula sp. 4* | v |  |  | Expert judge |
| *Navicula sp. 5* | v |  |  | Expert judge |
| *Nitzschia* cf. *gracilis* |  |  | v | Zindarova et al., 2016 |
| *Nitzschia* cf. *homburgiensis* |  |  | v | Witkowski et al., 2000 |
| *Nitzschia* cf. *wilmotteana* |  |  | v | Expert judge |
| *Nitzschia pellucida* | v |  |  | Witkowski et al., 2000 |
| *Nitzschia* sp. 1 | v |  |  | Expert judge |
| *Nitzschia* sp. 2 | v |  |  | Al-Handal and Wulff, 2008a |
| *Nitzschia* sp. 3 | v |  |  | Expert judge |
| *Nitzschia* sp. 4 | v |  |  | Witkowski et al., 2000; Buczkó et al., 2019 |
| *Nitzschia* sp. 5 | v |  |  | Expert judge |
| *Odontella litigosa* | v |  |  | Expert judge |
| *Paralia* sp. 1 | v |  |  | Expert judge |
| *Parlibellus crucicula* | v |  | v | Al-Handal and Wulff, 2008a |
| *Pinnularia australomicrostauron* |  |  | v | Round et al., 1990 |
| *Pinnularia* sp. 1 |  |  | v | Expert judge |
| *Pinnularia* sp. 2 |  |  | v | Al-Handal and Wulff, 2008a |
| *Planothidium* cf. *delicatulum* |  | v | v | Expert judge |
| *Pleurosigma* cf. *obscurum* | v |  | v | Expert judge |
| *Pleurosigma* sp. 1 | v |  |  | Expert judge |
| *Pseudogomphonema kamtschaticum* | v |  |  | Almandoz et al., 2014 |
| *Pseudogomphonema* sp. 1 | v |  |  | Al-Handal and Wulff, 2008b |
| *Pseudogomphonema* sp. 2 | v |  |  | Al-Handal and Wulff, 2008a |
| *Pteroncola carlinii* | v |  |  | Guiry and Guiry, 2020 (*AlgaeBase*) |
| *Rhabdonema arcuatum* | v |  | v | Expert judge |
| *Rhoicosphenia genuflexa* | v |  | v | Expert judge |
| *Rhoicosphenia* sp. 1 | v |  |  | Flower et al., 1996 |
| *Rhoicosphenia* sp. 2 | v |  |  | Cremer et al., 2003 |
| *Synedra* cf. *kerguelensis* | v |  |  | Cremer et al., 2003 |
| *Synedropsis laevis* | v |  |  | Cremer et al., 2003 |

**Table S8.** (Continued)

| **Species** | **Ecological type^*^** | | | **Reference** |
| --- | --- | --- | --- | --- |
|  | **m** | **eu** | **f** |  |
| *Synedropsis recta* | v |  |  | Cremer et al., 2003 |
| *Tabularia tabulata* | v | v | v | Al-Handal and Wulff, 2008b |
| *Thalassionema* cf. *nitzschioides* | v |  |  | Al-Handal and Wulff, 2008a |
| *Thalassiosira* cf. *punctigera* | v |  |  | Hasle and Syvertsen, 1996 |
| *Thalassiosira* sp. 1 | v |  |  | Expert judge |
| *Thalassiosira* sp. 2 | v |  |  | Expert judge |
| *Thalassiosira* sp. 3 | v |  |  | Expert judge |
| **Total (*n* = 92)** | **83** | **7** | **16** |  |

^*^Ecological type classified into marine (m), euryhaline (eu), and freshwater (f), based on the literature, except for some species of which ecological type was unknown; three criteria applied, 1) species only occurred in subtidal zone was allocated to the marine taxa, 2) species having morphological similarity to the known marine or freshwater was designated to the corresponding salinity range, and 3) primarily considered the observation of live diatoms in site-specific manner.

**Supplementary figures**


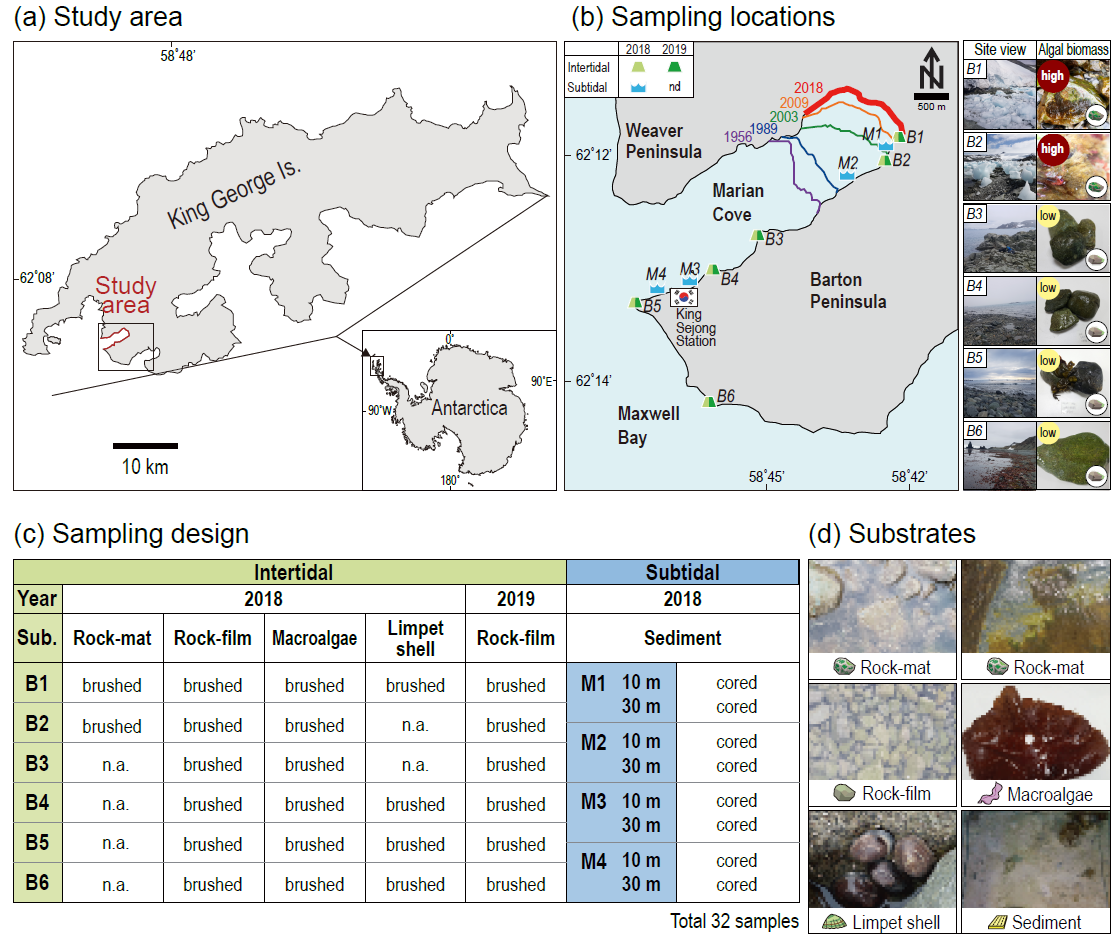
**Figure S1.** Map showing (a) the study area, (b) sampling locations (n = 10) with site views (B1–B6) and observed algal biomass. (c–d) sampling design was presented including information of substrate. The base maps were created in Adobe Illustrator CS6 based on the map of Google Earth (https://earth.google.com/web/).


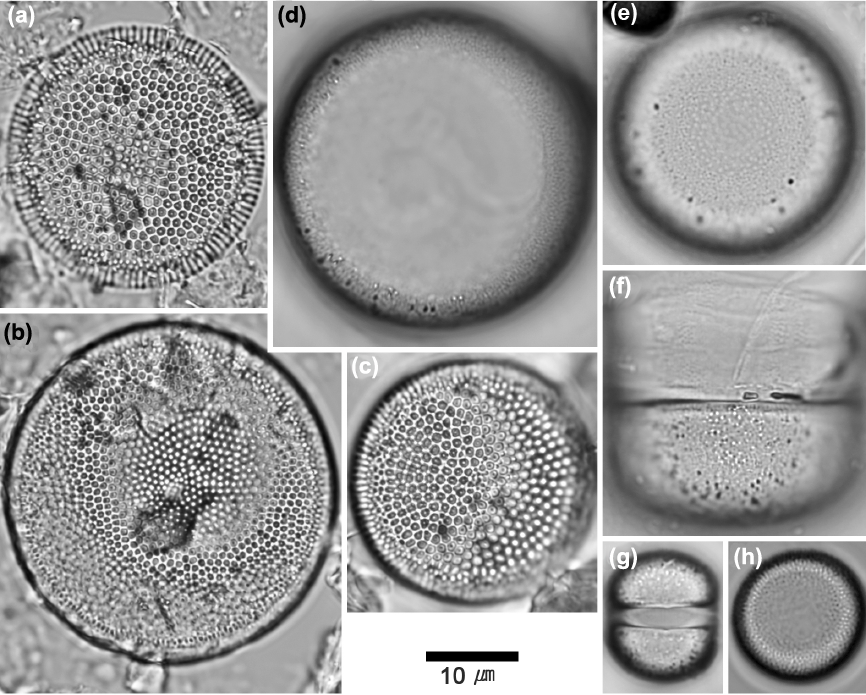


**Figure S2.** Light microscope photographs of centric diatoms in Marian Cove, West Antarctica. (**a–c**) *Actinocyclus* sp. 1; (**d–h**) *Melosira* sp. 1 (scale bar = 10 µm).


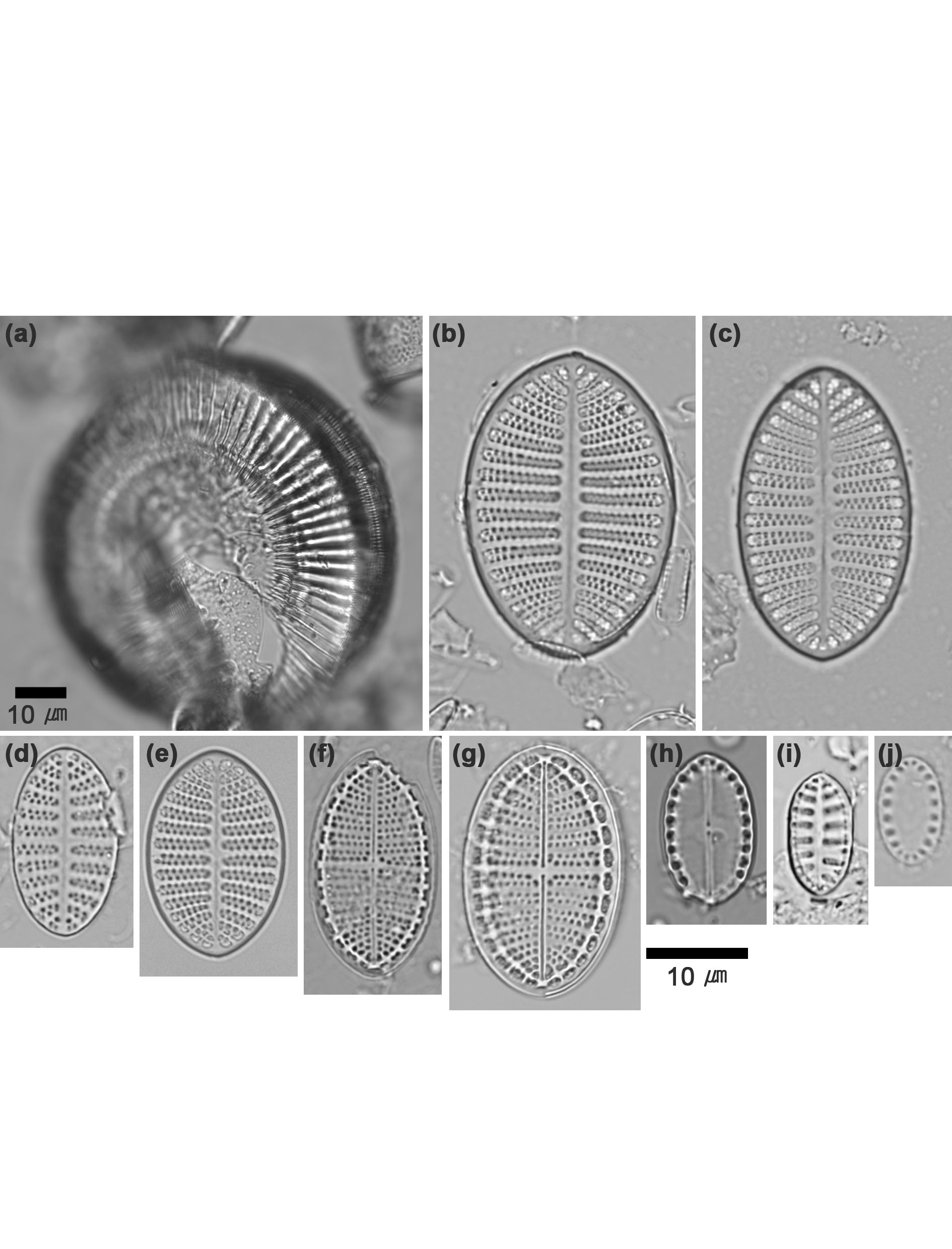
 **Figure S3.** Light microscope photographs of centric diatoms in Marian Cove, West Antarctica. (**a**) *Paralia* sp. 1; (**b–g**) *Cocconeis* cf. *pinnata*; (**h**) *Cocconeis pinnata* var. *matsii*; (**i–j**) *Cocconeis* *melchiroides* (scale bar = 10 µm).


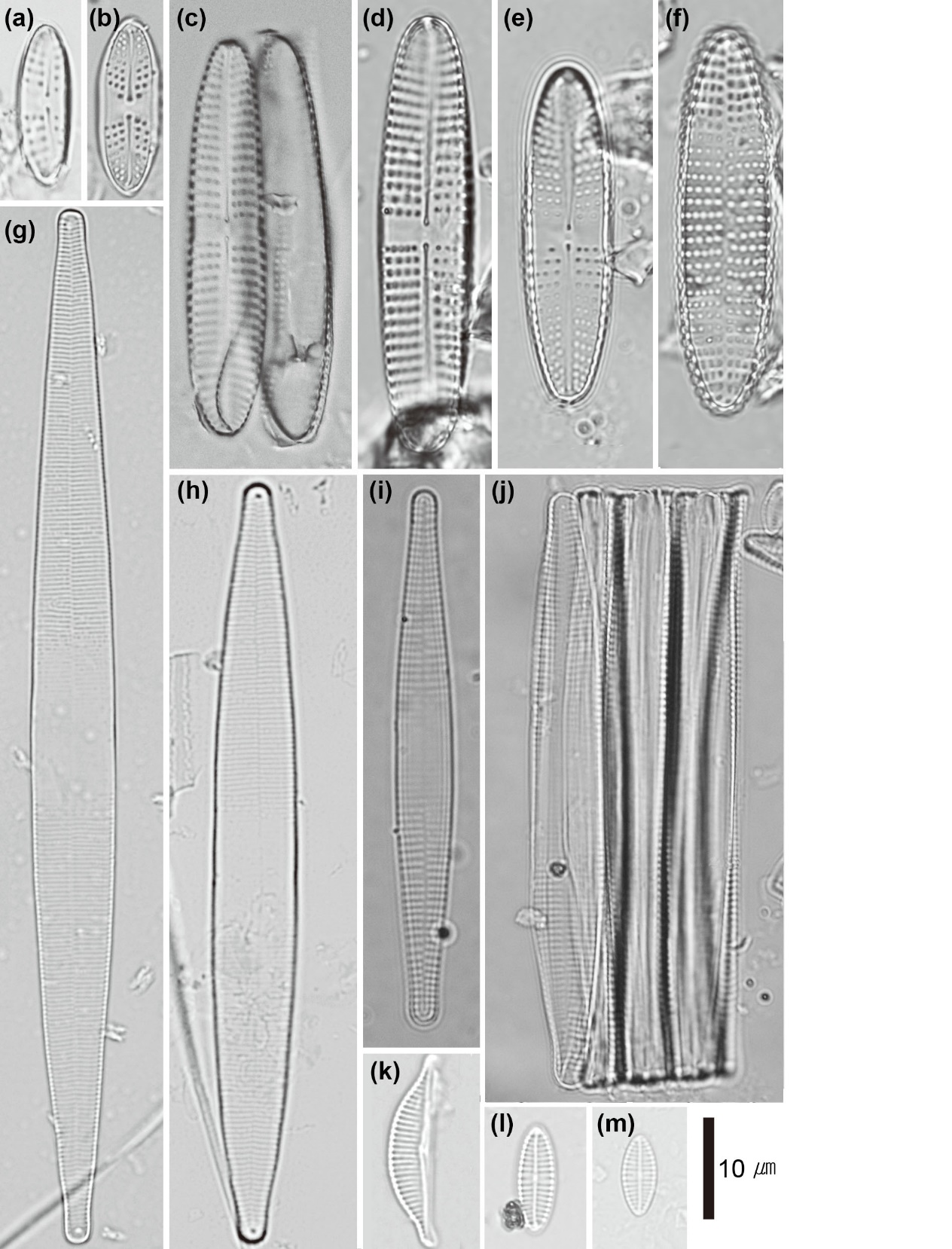


**Figure S4.** Light microscope photographs of pennate diatoms in Marian Cove, West Antarctica. (**a–b**) *Achnanthes* sp. 3; (**c–f**) *Achnanthes brevipes* var. *intermedia*; (**g–h**) *Brandinia mosimanniae*; (**i–j**) *Fragilaria striatula*; (**k**) *Halamphora* sp. 1; (**l–m**) *Navicula* sp. 5 (scale bar = 10 µm).


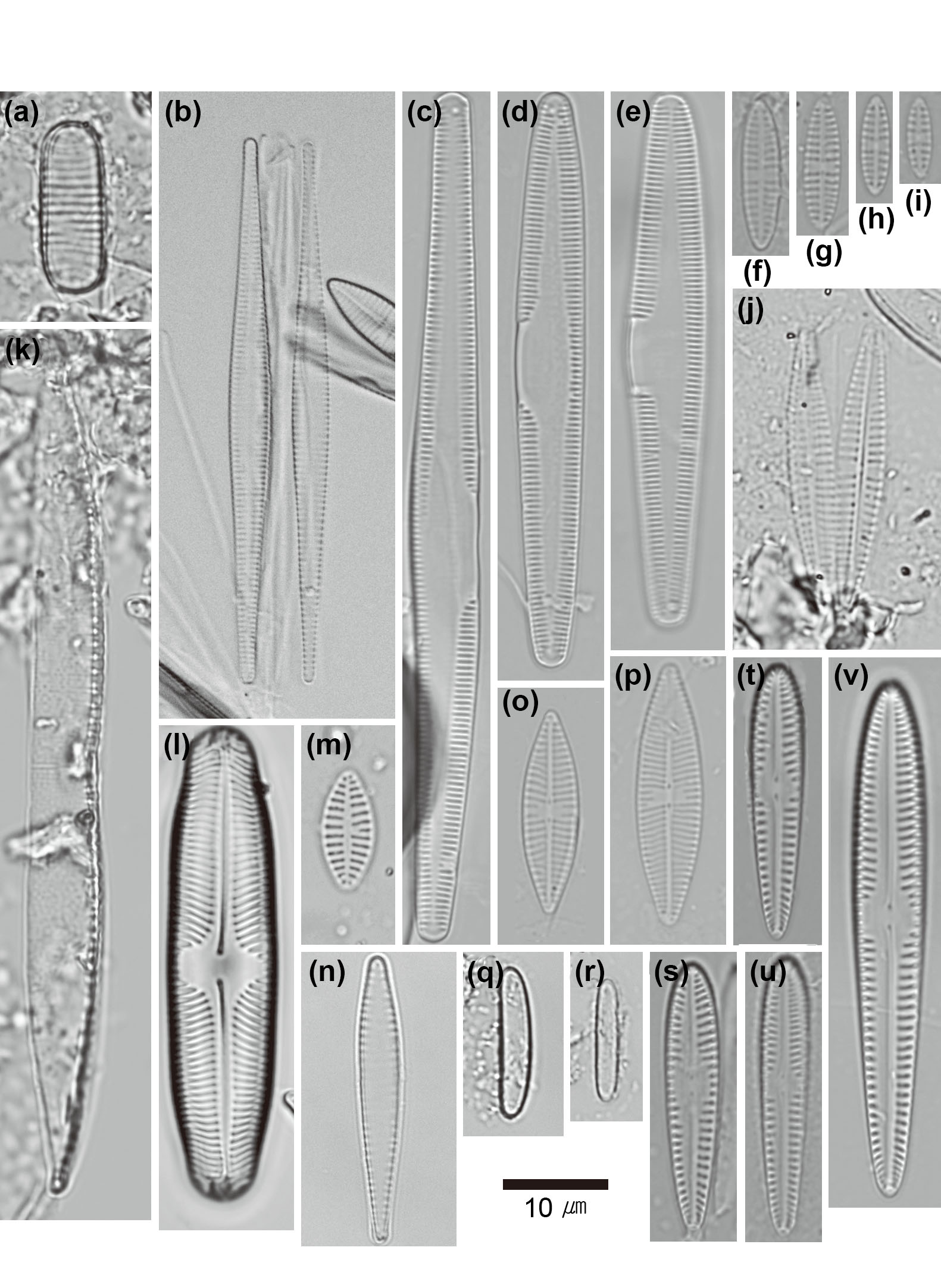


**Figure S5.** Light microscope photographs of pennate diatoms in Marian Cove, West Antarctica. (**a**) *Fragilariopsis* *curta*; (**b**) *Fragilaria islandica* var. *adeliae*; (**c–e**) *Tabulariopsis* cf. *australis*; (**f–i**) *Navicula* cf. *perminuta*; (**j**) *Navicula* cf. *directa*; (**k**) *Nitzschia* sp. 1; (**l**) *Pinnularia* *australomicrostauron*; (**m**) *Planothidium delicatulum*; (**n**) *Synedropsis* *recta*; (**o–p**) *Navicula glaciei*; (**q–r**) *Pteroncola carlinii*; (**s–u**) *Pseudogomphonema kamtschaticum* (scale bar = 10 µm).


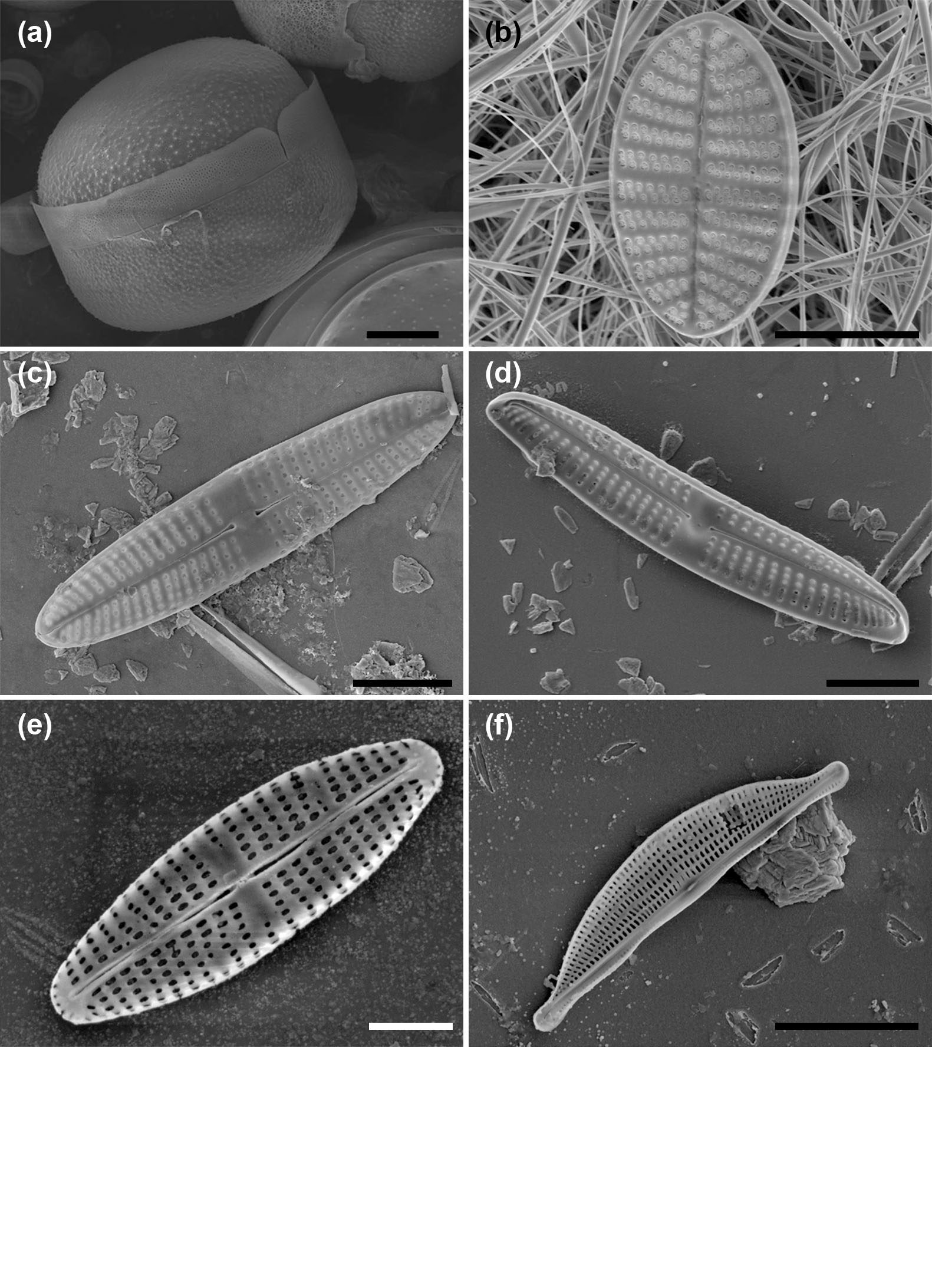
**Figure S6.** Scanning electron microscope (SEM) photographs of benthic diatoms in Marian Cove, West Antarctica. (**a**) *Melosira* sp. 1; (**b**) *Cocconeis* cf. *pinnata*; (**c–d**) *Achnanthes brevipes* var. *intermedia*; (**e**) *Navicula* sp. 5; (**f**) *Halamphora* sp. 1 (scale bar = 10 µm).


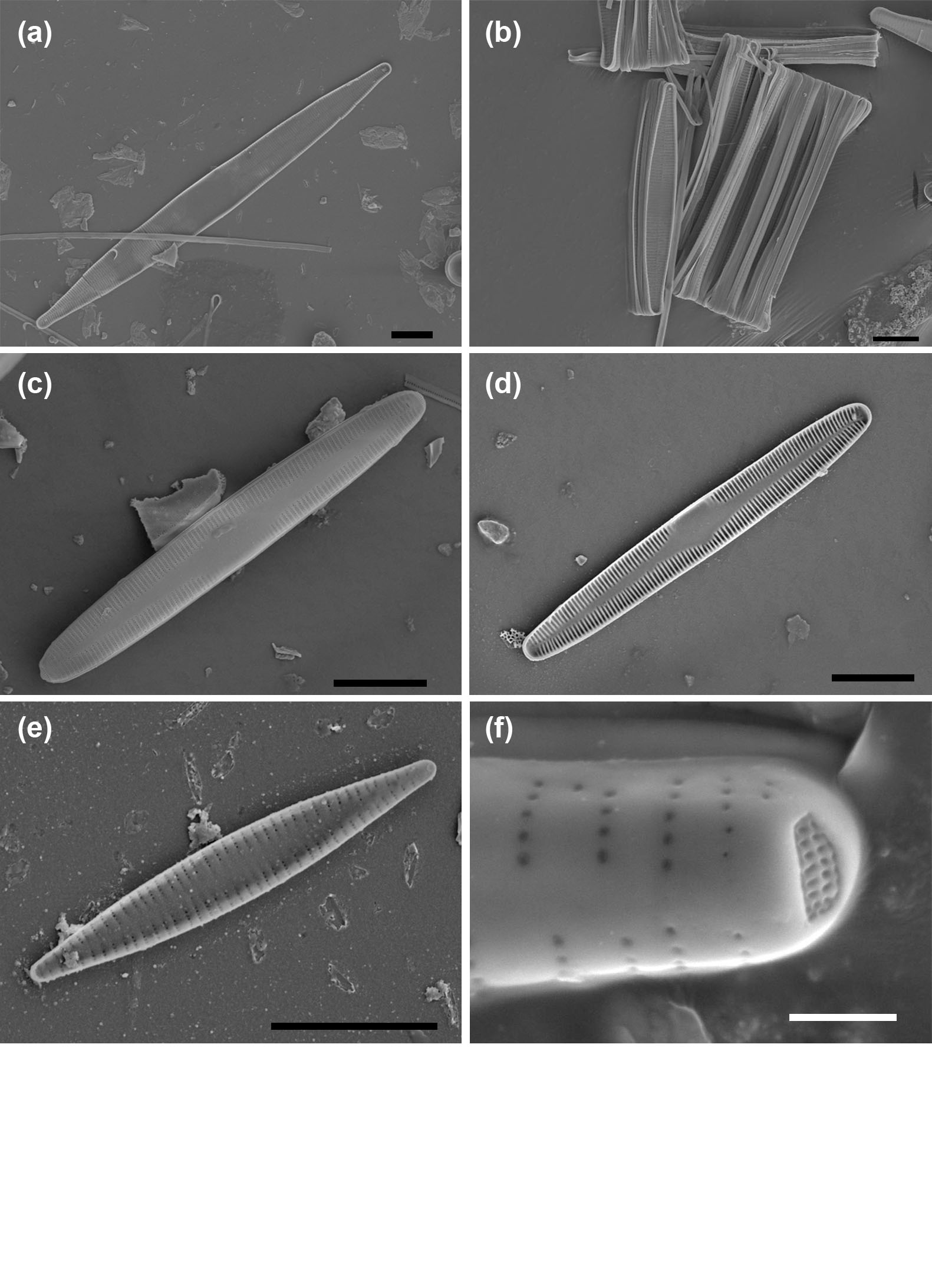
 **Figure S7.** Scanning electron microscope (SEM) photographs of benthic diatoms in Marian Cove, West Antarctica. (**a–b**) *Brandinia mosimanniae*; (**c–d**) *Tabulariopsis* cf. *australis*; (e**–f**) *Fragilaria islandica* var. *adeliae* (scale bar = 10 µm).


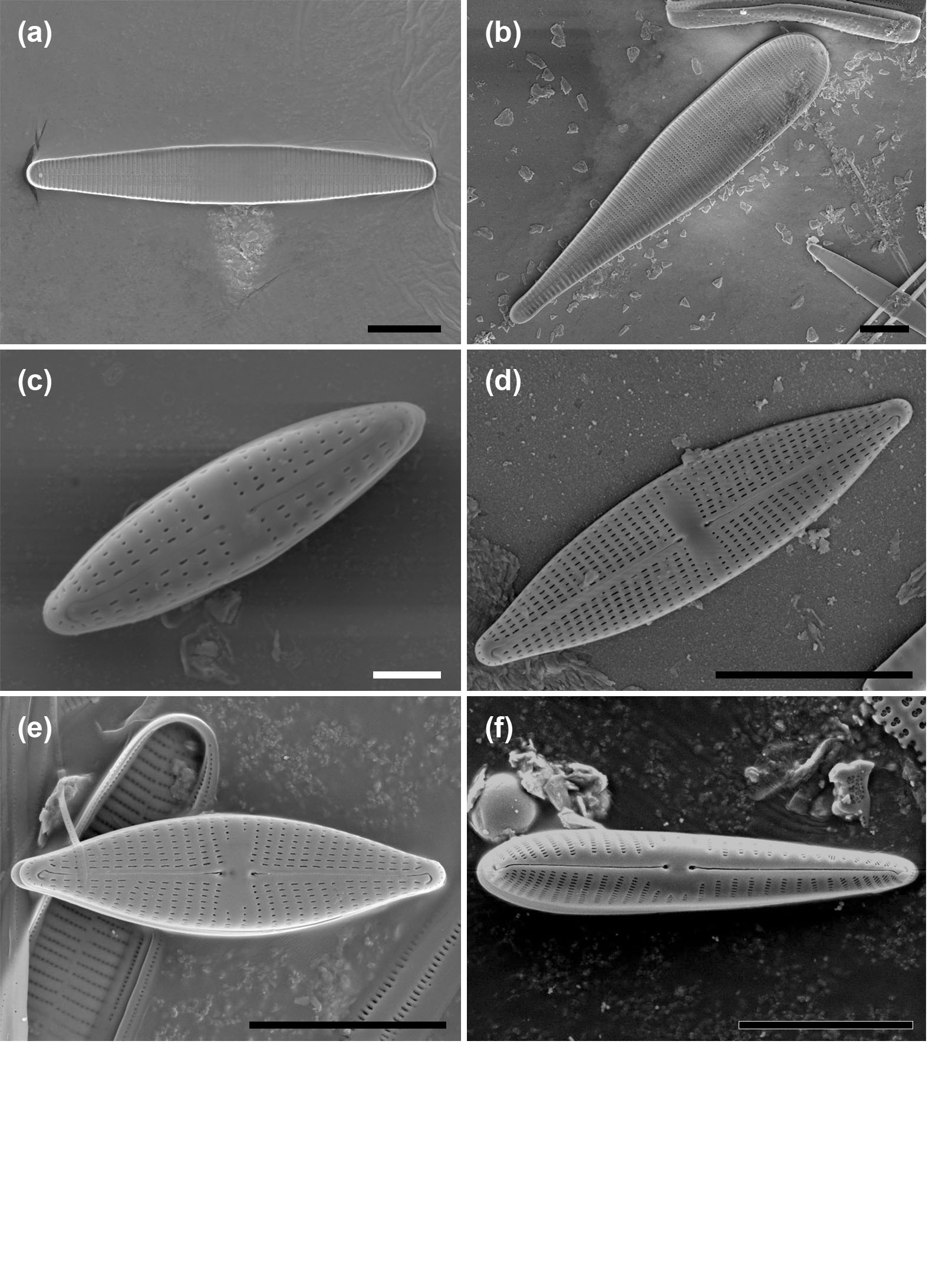
 **Figure S8.** Scanning electron microscope (SEM) photographs of benthic diatoms in Marian Cove, West Antarctica. (**a**) *Fragilaria* cf. *striatula*; (**b**) *Licmophora gracilis*; (**c**) *Navicula* cf. *perminuta*; (**d–e**) *Navicula glaciei*; (**f**) *Pseudogomphonema kamtschaticum* (scale bar in **a–b**, and **d–e** = 10 µm, and in c = 2 µm).


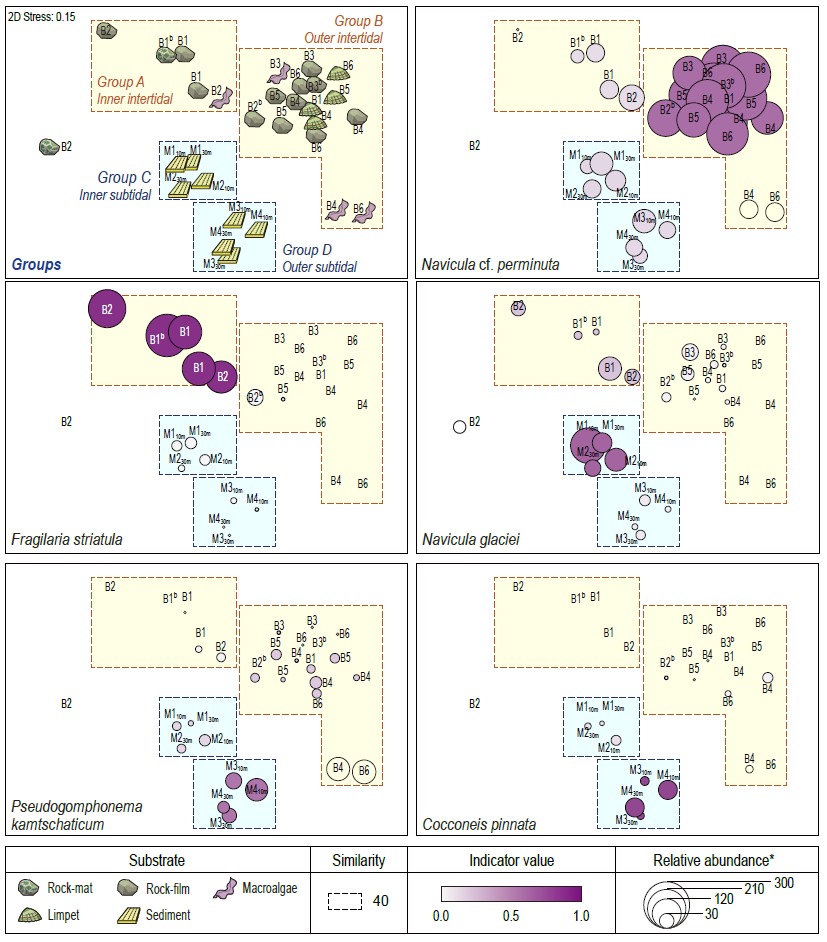


**Figure S9.** Illustration of the groups of benthic diatom assemblages in Marian Cove, West Antarctica, based on the NMDS (non-metric multidimensional scaling); five indicator species presented with corresponding indicator values and relative abundances (300 counts per sample).

**Supplementary references**

Al-Handal, A.Y. & Wulff, A. X. Marine benthic diatoms from Potter Cove, King George Island, Antarctica. *Botanica Marina*, 51, 51–68 (2008a).

Al-Handal, A.Y. & Wulff, A.J.B.M. Marine epiphytic diatoms from the shallow sublittoral zone in Potter Cove, King George Island, Antarctica. 51, 411–435 (2008b).

Almandoz, G.O., Ferrario, M.E., Sullivan, M.J., Ector, L. & Schloss, I.R. A new Pteroncola species (Bacillariophyceae) from the South Shetland Islands, Antarctica. *Phycologia*, 53, 188–194 (2014).

Buczkó, K., Ács, É., Báldi, K., Pozderka, V., Braun, M., Kiss, K.T. *et al.* The first high resolution diatom record from Lake Balaton, Hungary in Central Europe. *Limnetica*, 38, 417–430 (2019).

Cremer, H., Roberts, D., McMinn, A., Gore, D. & Melles, M. The Holocene diatom flora of marine bays in the Windmill Islands, East Antarctica. *Botanica Marina*, 46, 82–106 (2003).

Fernandes, L.F., Calixto-Feres, M., Rivera Tenenbaum, D., Procopiak, L.K., Portinho, D. & Hinz, F. Fine morphology of four *Licmophora* (Bacillariophyta, Licmophorales) species from Admiralty Bay and Elephant Island, Antarctic Peninsula. *Iheringia, Sér. Bot.*, 69, 465–477 (2014).

Fernandes, L.F., Procopiak, L.K. & Portinho, D. *Brandinia mosimanniae* gen. nov. et sp. nov., a new marine epilithic diatom from the Antarctic coasts. *Diatom Research*, 22, 45–56 (2007).

Flower, R.J., Jones, V.J. & Round, F.E. The distribution and classification of the problematic *Fragilaria* (virescens v.) *exigua* Grun./*Fragilaria exiguiformis* (Grun.) Lange-Bertalot: a new species or a new genus? *Diatom Research*, 11, 41–57 (1996).

Guiry, M.D. & Guiry, G.M. World-wide electronic publication, National University of Ireland, Galway. *AlgaeBase*. http://www.algaebase.org; searched on 25 February 2020.

Hasle, G.R. & Syvertsen, E.E. Marine diatoms. In: Identifying Marine Phytoplankton. (Tomas, C.R. Eds), 5–385. Academic Press (1996).

Hendey, N.I. A revised check-list of British marine diatoms. *Journal of the Marine Biological Association of the United Kingdom*, 54, 277–300 (1974).

Lange-Bertalot, H., Hofmann, G., Werum, M. & Cantonati, M. Freshwater benthic diatoms of Central Europe: over 800 common species used in ecological assessments. 1–942. Schmitten-Oberreifenberg: Koeltz Botanical Books (2017).

Trabert, Z. Diatoms from the Antarctic region: Maritime Antarctica. *Acta Botanica Hungarica*, 59, 277–279 (2017).

Zindarova, R., Kopalová, K. & Van der Vijver, B. Diatoms from the Antarctic region: maritime Antarctica. Bibliotheca Diatomologica 28, 9–504 (2016).
